# Multi-scale spatial genetic structure of the vector-borne pathogen ‘*Candidatus* Phytoplasma prunorum’ in orchards and in wild habitats

**DOI:** 10.1038/s41598-020-61908-0

**Published:** 2020-03-19

**Authors:** Véronique Marie-Jeanne, François Bonnot, Gaël Thébaud, Jean Peccoud, Gérard Labonne, Nicolas Sauvion

**Affiliations:** 1 BGPI, Univ Montpellier, INRAE, CIRAD, Institut Agro, Montpellier, France; 2 Present Address: Université de Poitiers, Laboratoire Ecologie et Biologie des Interactions, (EBI—Joint Research Unit 7267, CNRS), 86000 Poitiers, France

**Keywords:** Entomology, Ecology, Ecological epidemiology, Ecological networks

## Abstract

Inferring the dispersal processes of vector-borne plant pathogens is a great challenge because the plausible epidemiological scenarios often involve complex spread patterns at multiple scales. The spatial genetic structure of ‘*Candidatus* Phytoplasma prunorum’, responsible for European stone fruit yellows disease, was investigated by the application of a combination of statistical approaches to genotype data of the pathogen sampled from cultivated and wild compartments in three French *Prunus*-growing regions. This work revealed that the prevalence of the different genotypes is highly uneven both between regions and compartments. In addition, we identified a significant clustering of similar genotypes within a radius of 50 km or less, but not between nearby wild and cultivated *Prunus*. We also provide evidence that infected plants are transferred between production areas, and that both species of the *Cacopsylla pruni* complex can spread the pathogen. Altogether, this work supports a main epidemiological scenario where ‘*Ca*. P. prunorum’ is endemic in — and generally acquired from — wild *Prunus* by its immature psyllid vectors. The latter then migrate to shelter plants that epidemiologically connect sites less than 50 km apart by later providing infectious mature psyllids to their “migration basins”. Such multi-scale studies could be useful for other pathosystems.

## Introduction

Vector-borne pathogens have caused some of the most devastating plant diseases in perennial and annual crops^1,2^. Managing these threats requires an understanding of their epidemiological cycle, particularly the dispersal processes of pathogens in the landscape^3^. However, deciphering the complexity of plausible epidemiological scenarios poses a great challenge, especially since insects are involved in disease spread. Indeed, most of the vector-borne plant pathogens are transmitted by small piercing-sucking insects of the Hemiptera order, whose dispersal distances are generally only vaguely known, while searching host plants for food resources and/or reproduction^4^. Added to this question of dispersal distance is the issue of different host plant species that insects can occupy during their life cycle and how they move among them. In this context, a key question that often arises is the role of wild plants as a reservoir of pathogen and/or insect vectors^5–10^.

One of the first difficulties in identifying patterns of disease spread in agricultural landscapes is to determine the optimal scale of the study design. Although the spatial scale of the processes has been a central question for decades in ecology^11–14^ and population genetics^15,16^, the development of large-scale pattern-oriented approaches to understand the processes that shape the genetic structure of a population is recent^17–21^. The basic idea of these approaches is to estimate the distance at which two samples become genetically independent by linking genetic data and spatial information obtained for a set of samples using a combination of geostatistics^22^ and population or landscape genetics methods^23^. In particular, approaches used for qualitative data comprise join counts^24^ and permutation tests to identify distances at which genetic similarity between pairs of individuals is significantly higher than expected by chance^25–28^.

The present study focuses on a fruit tree disease known as European stone fruit yellows (ESFY), caused by ‘*Candidatus* Phytoplasma prunorum’^29^ and disseminated via planting material^30^ or the psyllid *Cacopsylla pruni* (Scopoli, 1763)^31^. The phytoplasma and its psyllid vectors are widespread in Europe, including the major stone fruit production areas where the disease significantly impacts susceptible crops, particularly apricot and Japanese plum trees^30,32^. Rigorous sanitary control of nursery plants and insecticide treatments are currently the main disease-control strategies, but despite these measures, ESFY continues to economically impact European fruit growers, raising the question of the origin of contaminations.

**Figure 1:**
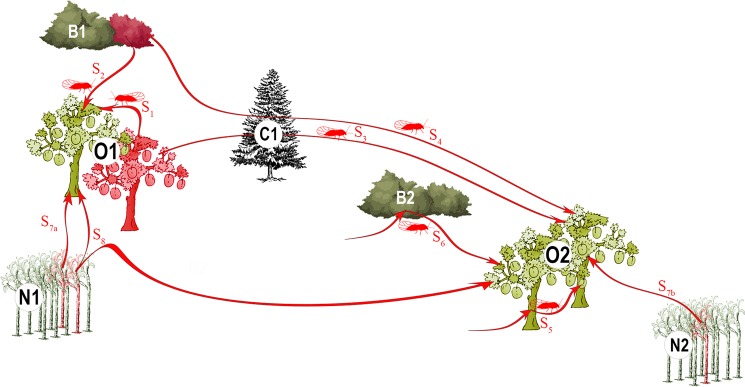
Eight mutually non-exclusive eco-epidemiological scenarios through which ‘*Ca*. P. prunorum’ might be spread in orchards. Bi: bushes (i.e., wild *Prunus* = host plants); Ci: conifers (i.e., shelter plants); Ni: nurseries; Oi: orchards. In red: cultivated trees, nursery plants and wild *Prunus* infected by the phytoplasma or infectious psyllids; in green: non-infected plants. Pathogen transmission to cultivated trees may involve various spatio-temporal scales depending on the scenario involved: transmission to a healthy apricot tree by a psyllid that acquired the pathogen from a nearby infected cultivated tree (S1) or a nearby infected bush (S2); transmission by a mature psyllid that acquired the phytoplasma on an infected tree (S3) or bush (S4) the previous year; multiple transmissions by the same infectious psyllid to nearby cultivated trees (S5) or to a bush and then a nearby cultivated tree (S6); independent contaminations of orchards by plants from nearby nurseries (S7a + S7b); contaminations of distant orchards by plants from the same nursery (S8). Figure was generated with Photoshop CS6 (https://www.adobe.com/products/photoshop.html).

Over the past 20 years, several studies have aimed to decipher the complexity of the phytoplasma/*Prunus*/psyllid pathosystem^33^. Two points appear essential in the dynamics of the epidemic: (i) in Europe, the pathogen is often found in wild *Prunu*s^32,34^; and (ii) the psyllid vectors are univoltine and migrate twice a year between *Prunus* and conifers^9^. In a landscape comprising different plants on which the psyllid vectors can feed, different mutually non-exclusive scenarios of pathogen spread in orchards can be proposed (Fig. 1). Thébaud *et al*.^9^ showed that the most likely path of orchard contamination involved mature (i.e., immigrant) psyllids carrying the phytoplasma at a sufficient concentration for inoculation (e.g., scenarios 3 and 4 in Fig. 1). Nevertheless, this lab-based conclusion has not yet been confirmed by field studies that investigate the role played by the local secondary spread of ESFY by immature (i.e., emigrant) or mature (i.e., immigrant) *C. pruni* involved in either within-orchard tree-to-tree transmission (e.g., scenario 1 in Fig. 1) or acquisition of the phytoplasma in bushes before transmission to a nearby orchard (e.g., scenario 2 in Fig. 1). The relative contribution of natural spread and human transfer of contaminated plants is another major unknown in this pathosystem (scenarios 7_a+b_ and 8 in Fig. 1). Finally, the spatial scale of these processes is unknown and, in combination with the landscape structure, this may lead to more or less complex epidemiological networks that connect close or distant ecological compartments (Supplementary Fig. S1).

The goal of the present work is to improve our understanding of the spatial scale and ecological compartments involved in ESFY epidemics by investigating the spatial genetic structure of ‘*Ca*. P. prunorum’ using complementary statistical approaches that combine genetic data and geographical distances. Our study was conducted in three French growing regions [Pyrénées-Orientales (PO), Bas-Rhône (BR) and Valence (VA)] with different agroecological conditions and strongly impacted by the disease. There, we intensively sampled the three main hosts (psyllids, wild and cultivated *Prunus*) of the pathogen.

## Results

### Genetic diversity of ‘*Ca*. phytoplasma prunorum’

In the three sampling regions, we collected: (i) plant samples in infected orchards (mainly apricot); (ii) plant samples in wild *Prunus* bushes (mainly blackthorn) at various distances from these orchards; and (iii) mature psyllids in these bushes (Supplementary Figs. S2, S3, S4 and S5). A total of 6,342 samples were collected and tested for the presence of ‘*Ca*. P. prunorum’ (Table 1). The prevalence of ‘*Ca*. P. prunorum’ in bushes showed that: on average, 43.3% of the samples tested positive, from which we obtained 339 sequences of the immunodominant membrane protein (*imp*) gene. From the 69 infected orchards, 750 samples tested positive for the phytoplasma, and 553 *imp* sequences were obtained. In the 15 orchards where a systematic sampling was undertaken, 14.3% of the 1,982 sampled trees tested positive for the phytoplasma (Supplementary Table S1), and only 4.3% of these were asymptomatic. Among 117 samples from symptomatic trees sampled several times (at different times or on different branches at the same date), six showed distinct *imp* genotypes; in this case different branches of the same tree were considered as different samples. Among the 2,572 psyllids collected from 71 different bushes, 104 (4.8%) were found to carry the phytoplasma, from which we obtained 99 sequences. Thus, 991 samples were successfully genotyped for the *imp* gene (Table 1).

**Table 1:** Statistics on the samples from each of the three ecological compartments (bush, psyllid and orchard), all regions combined and within each region.

Region	Compartment	n_1_	n_2_ [min-max]	N	#ESFY +	%ESFY + [min-max]	#IMP
PO + BR + VA	bush	61	21 [5–46]	1114	474	43.3 [0–100]	339
	psyllid	71	34 [1–196]	2572	104	4.8 [0–50.0]	99
	orchard	69	37 [1–244]	2656	750	13.0 [1.9–39.3]	553
	∑	201		6342	1328		991
PO	bush	14	24 [10–46]	332	163	47.4 [0–100]	123
	psyllid	32	54 [7–196]	1723	61	3.5 [0.7–14.3]	58
	orchard	34	28 [1–255]	953	331	17.1 [1.9–39.3]	267
	∑	80		3008	555		448
BR	bush	33	13 [5–40]	426	152	39.2 [0–100]	99
	psyllid	11	27 [2–58]	299	19	6.4 [1.9–50.0]	19
	orchard	24	38 [1–140]	901	254	12.5 [4.0–25.9]	164
	∑	68		1626	425		282
VA	bush	14	25 [16–33]	356	159	43.4 [0–100]	117
	psyllid	28	20 [1–76]	550	24	4.4 [0–50.0]	22
	orchard	11	44 [2–244]	802	165	9.3 [2.7–19.3]	122
	∑	53		1708	348		261

PO: Pyrénées-Orientales; BR: Bas-Rhône; VA: Valence. n_1_: number of orchards or bushes per region in which plant or insect samples were collected. n_2_: mean number of plant samples (or psyllids) collected per orchard or bush [minimum and maximum values]. N: total number of samples collected per region. #ESFY + : total number of samples found to be positive by PCR. %ESFY + : mean percentage of PCR-positive plant samples (or psyllids) per orchard or bush [minimum and maximum values]. #IMP: number of samples where the *imp* gene was successfully genotyped. ∑: sum.

**Figure 2:**
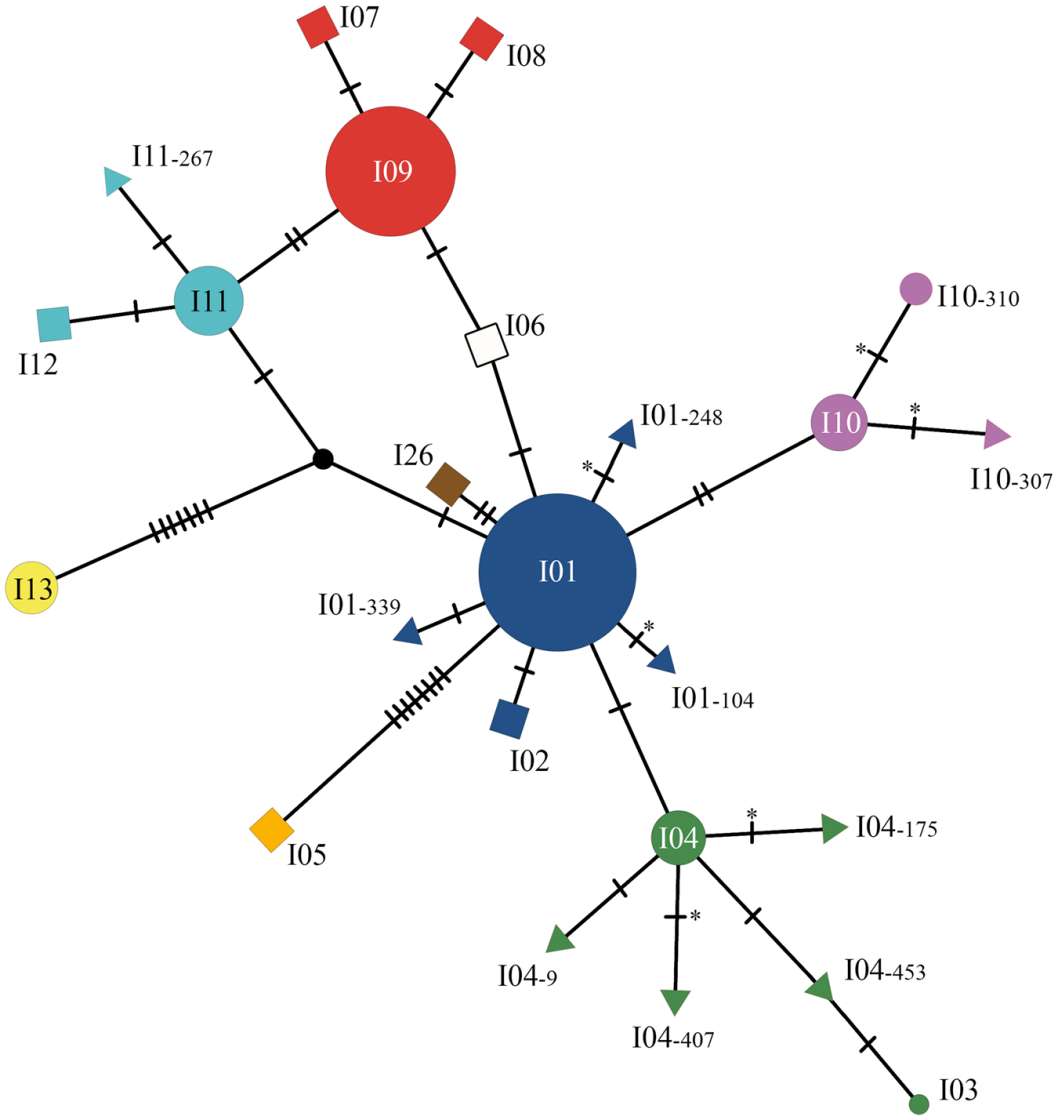
Genotype network inferred from 24 *imp* sequences of ‘*Ca*. P. prunorum’ using the integer neighbour-joining algorithm. The disks represent the previously described genotypes that were found in our samples. Disk area is proportional to the number of genotyped samples (Supplementary Table S2). The squares represent the previously described genotypes that were not found in our samples. The triangles represent the newly described genotypes. Each of them was named according to the major genotype from which it was derived, followed by the position of the mutation in the open reading frame of the *imp* DNA sequence. Hatch marks along edges represent the number of mutations differentiating two connected sequences. Asterisks indicate changes in the amino acid sequence. The black node represents an inferred unsampled sequence. Network was generated with POPART software^52^ (v.1.7), and the figure was finalized with Photoshop CS6 (https://www.adobe.com/products/photoshop.html).

After sequencing, we identified 17 *imp* genotypes where genotypes I01 and I09 were the most prevalent (44.2% and 30.0% of the samples, respectively; Supplementary Table S2). Four of other *imp* genotypes (i.e., I04, I10, I11, I13) reached a frequency above 4%. Thus, only these six major genotypes were considered in the subsequent statistical analyses. Among the 11 other genotypes, I03 had already been described in Germany by Danet *et al*.^35^, and I10–310 was recently described in Slovenia by Dermastia *et al*.^36^ under the name I34. Nine genotypes (i.e., I01–104, I01–248, I01–339, I04–9, I04–175, I04–407, I04–453, I10–307 and I11–267) had never been described before (Supplementary Tables S2 and S6). Conversely, five genotypes (I02, I06, I07, I08, I12) previously described in France by Danet *et al*.^35^ were not found in our survey. The network illustrating the genetic relationships among the genotypes shows that most of the genotypes obtained differ by only one or two single nucleotide polymorphisms (SNPs), whereas genotypes I13 and I05 widely differ from all the other genotypes (Fig. 2).

The psyllid vector *C. pruni* is known to present two cryptic species currently referred to as A and B, which show clear genetic differences despite being ecologically and morphologically indistinguishable^37,38^. All the collected psyllids were successfully assigned to either species. Among all of the regions combined, species A was more frequent (62.6% of the samples; Supplementary Table S5), but the proportion greatly varies depending on the region, particularly in BR where this species strongly prevails (97.3%). There was no significant difference in phytoplasma prevalence (grey cells in Supplementary Table S5) between psyllid species across all regions (*p* = 0.52; Fisher’s exact test). The proportions of the six major genotypes significantly differed between the two species (*p* = 2 × 10^−4^ across all regions; Fisher’s exact test), mainly because of the over-representation of genotype I01 in species B. For the following analysis, the *C. pruni* complex was considered as a single epidemiological entity because performing distinct analyses for species A and B strongly reduced the statistical power.

### Exploratory data analysis

**Figure 3:**
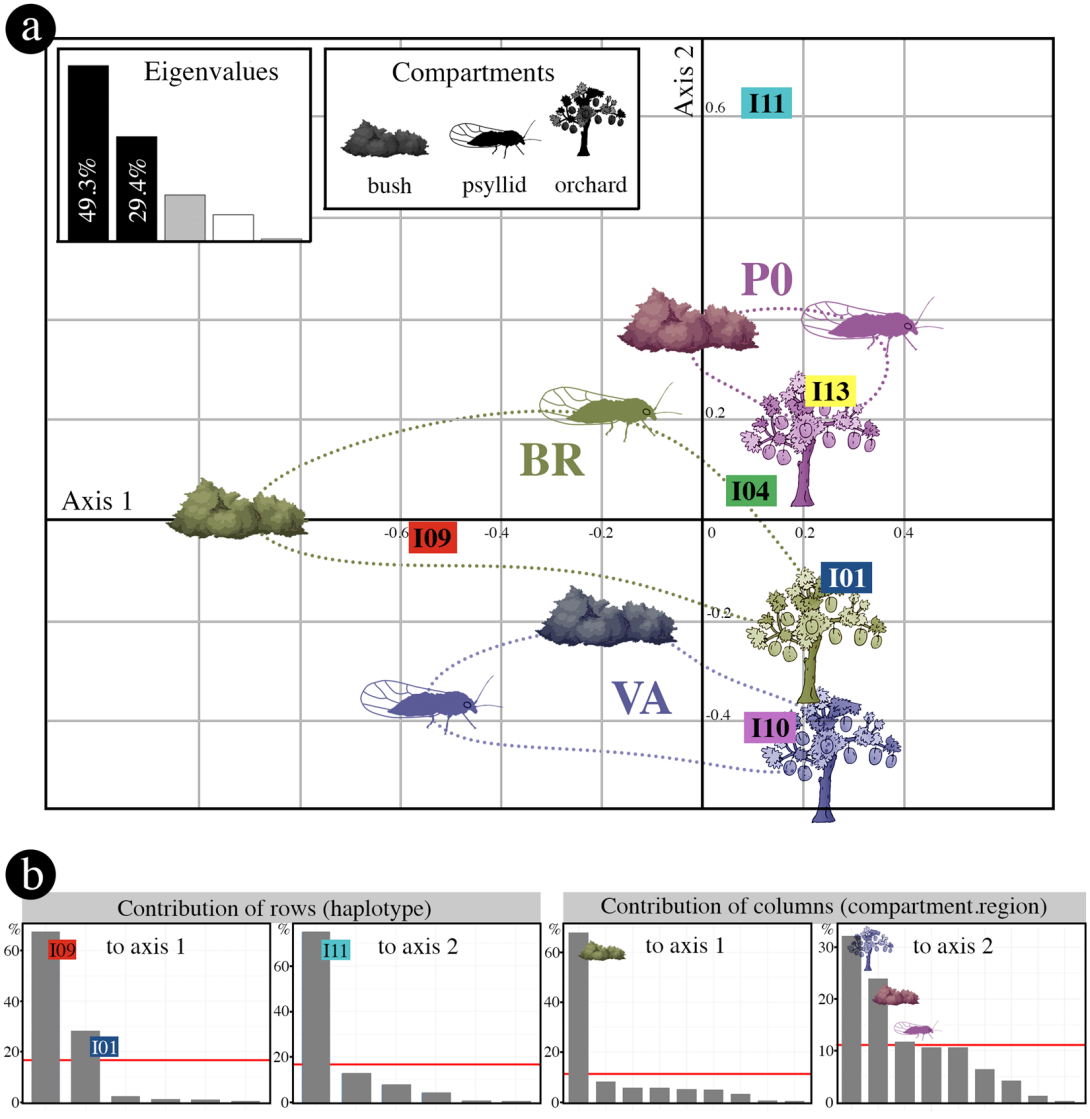
Correspondence analysis performed on the contingency table displaying the frequency distribution of the six major genotypes in the three ecological compartments and the three regions. (**a**) Factorial map of the first two axes. Each compartment is represented by its symbol; the associated colours and ellipses are interpretation aids that make it possible to visualise the modalities associated with each region. The eigenvalue plot illustrates the percentage of variance explained by the different axes. (**b**) Contributions of rows and columns of the contingency table to the first two dimensions of the analysis. The red line represents the expected row or column contributions if the contributions were uniform. Any row or column above this threshold is a major contributor to the corresponding axis. Factorial map (**a**) and graphs illustrating the contributions of rows and columns (**b**) were generated with statistical software R 3.4.0^53^, respectively with *coa* function in the *ade4* package^54^ and *factoextra* package^55^. Then, the figure was finalized with Photoshop CS6 (https://www.adobe.com/products/photoshop.html).

In order to explore relationships among genotypes, compartments and regions, we performed a correspondence analysis on the contingency table of genotype frequencies (Supplementary Table S3). Since the first two axes of the correspondence analysis explain a large part (78.7%) of the variance (Fig. 3), the dimension reduction obtained can be considered as very acceptable to interpret the data. The main modalities contributing to axis 1 are genotype I09 and BR bushes and, to a lesser extent, genotype I01, while the main modalities contributing to axis 2 are genotype I11, VA orchards and PO bushes (Fig. 3b). In the PO region, all compartments appear to be very closely related and well separated from those of the VA region. The BR region has an intermediate profile and the three compartments were dissimilar compared to other regions (Fig. 3a). In addition, the genotypes from orchards were genetically more closely related (i.e., segregating only on axis 2) than those of the other two compartments, which hints at orchard-specific processes.

**Figure 4:**
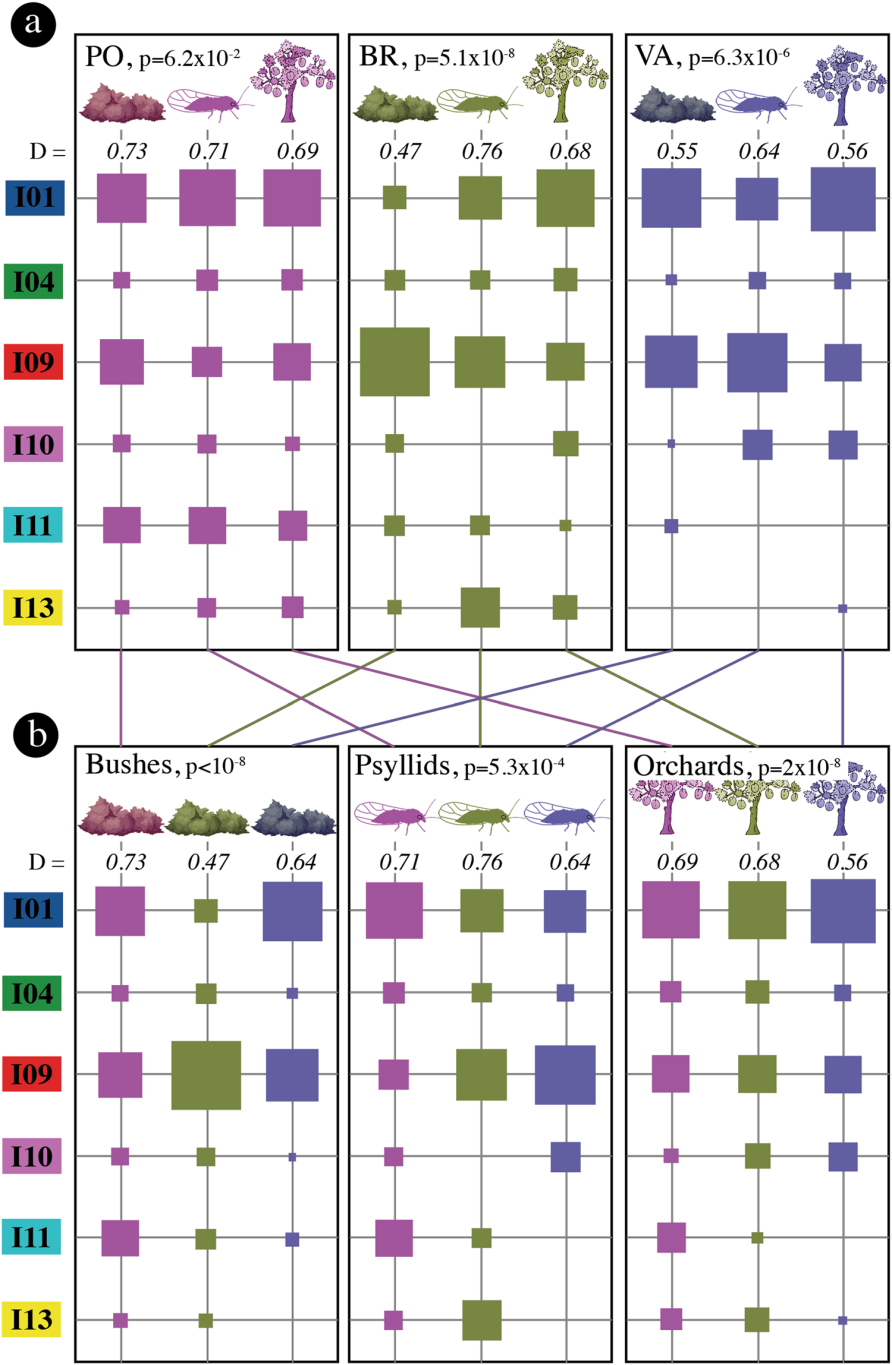
Relative proportions of the six major genotypes in each of the three ecological compartments. The same data are either (**a**) grouped by region (PO: Pyrénées-Orientales; BR: Bas-Rhône; VA: Valence), or (**b**) grouped by ecological compartment. P-values correspond to Fisher’s exact test that determines if genotype profiles are identical either within a given region (in a), or within a given ecological compartment (in b). The values of Simpson’s diversity index D are italicised. The figure was generated using the *table.value* function in *ade4* package^54^, and finalized with Photoshop CS6 (https://www.adobe.com/products/photoshop.html).

To assess the significance of the observed patterns (Fig. 3a), we first carried out multinomial logistic regressions, which revealed a highly significant interaction (*p* = 6.5 × 10^−7^; likelihood ratio test) between compartments (i.e., bush, psyllid, orchard) and regions (i.e., PO, BR, VA). Consequently, we further studied these interactions by comparing the distributions of genotypes between compartments for each region (Fig. 4a) and the distributions of genotypes between regions for each compartment (Fig. 4b). In the PO region, there was no statistically significant difference (*p* = 0.062; Fisher’s exact test) between the visually homogeneous compartment profiles with very close values of Simpson’s diversity index D (Fig. 4a). This region was characterised by a high prevalence of genotype I11 in all three compartments, whereas it seemed less abundant or absent in the compartments of the other two regions (Fig. 4a; Supplementary Tables S3 and S4). The BR region shows very contrasted genotype frequency profiles among compartments (*p*  = 5.1 × 10^−8^; Fisher’s exact test) and strong differences between the values of the diversity index D. The high prevalence of genotype I09 in the bushes and, conversely, the high prevalence of genotype I01 in the orchards contribute to this highly significant genotype dissimilarity (Fig. 4a; Supplementary Tables S3 and S4). Within the VA region, the contrasted genotypic composition (*p* = 6.3 × 10^−6^; Fisher’s exact test) is also reflected by the higher value of the diversity index among psyllids than among bushes or orchards. Within each compartment, the genotypic composition significantly differed according to the growing region (Bushes: *p* < 10^−8^; Psyllids: *p* = 5.3 × 10^−4^; Orchards; *p*  = 2 × 10^−8^), although the proportion of the main genotypes for the orchards was very similar across regions (Fig. 4b), as mentioned above (Fig. 3b).

### Spatial patterns

In order to assess whether the sampled genotypes were spatially structured within and between compartments, the relationship between genetic and geographical distances was tested at all distances using a geostatistical method^24^. These spatial analyses were performed independently within each region because the above-mentioned interaction between compartments and regions meant that the genotype frequency distribution among compartments differed between the three growing regions.

**Figure 5:**
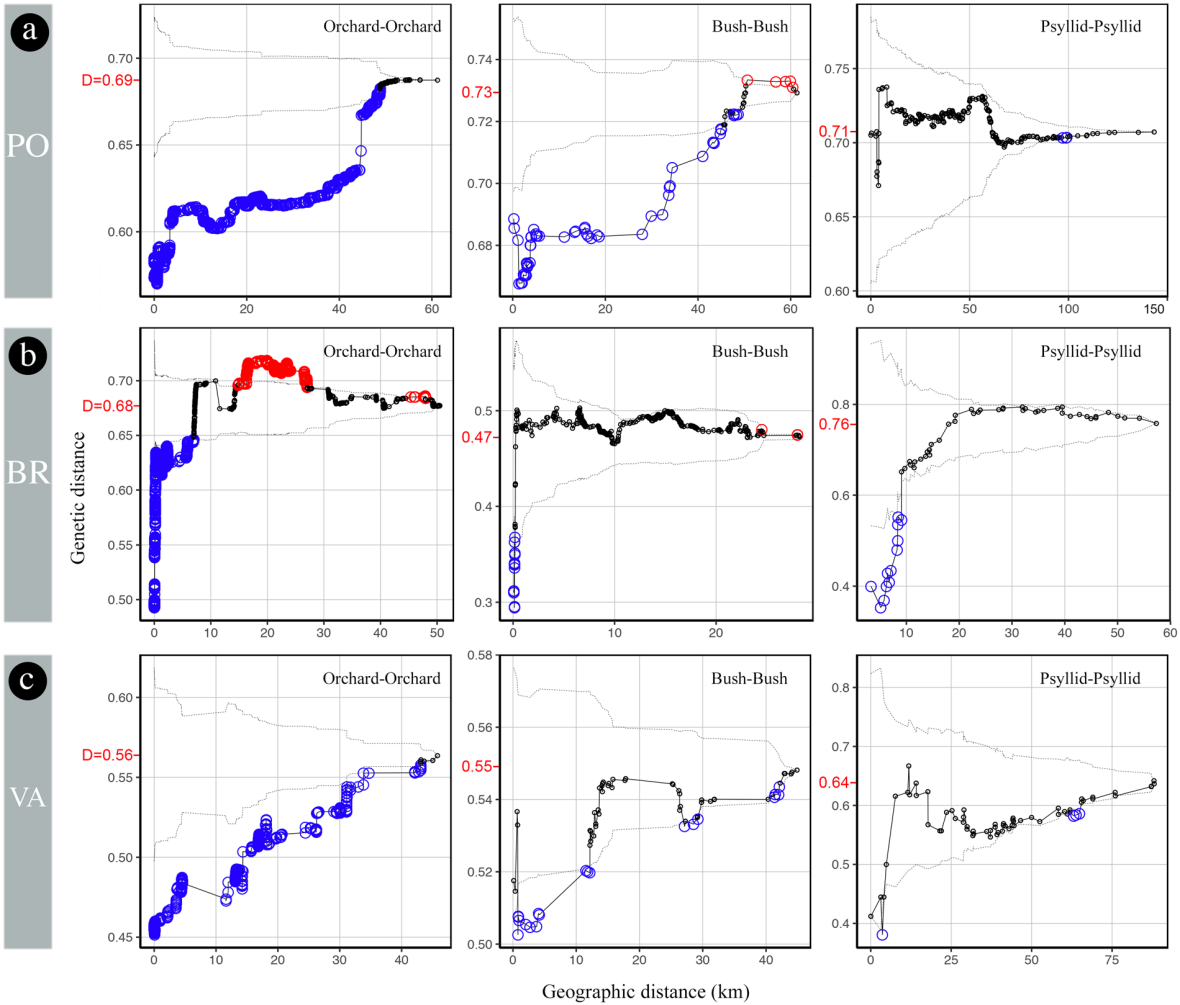
Mean pairwise genetic distances within increasing radii computed for all pairs of samples from each compartment in the (**a**) Pyrénées-Orientales, (**b**) Bas-Rhône, and (**c**) Valence regions. Solid line, observed values; dashed lines, 95% confidence envelope under the null hypothesis of spatial independence. Bigger circles below (resp., above) the envelope indicate radii within which the mean pairwise genetic distance is significantly lower (resp., higher) than expected in the absence of geographical structure. The values of Simpson’s diversity index D are in red on the *y*-axis. Graphs were generated using the statistical software R 3.4.0^53^, and the figure was finalized with Photoshop CS6 (https://www.adobe.com/products/photoshop.html).

**Figure 6:**
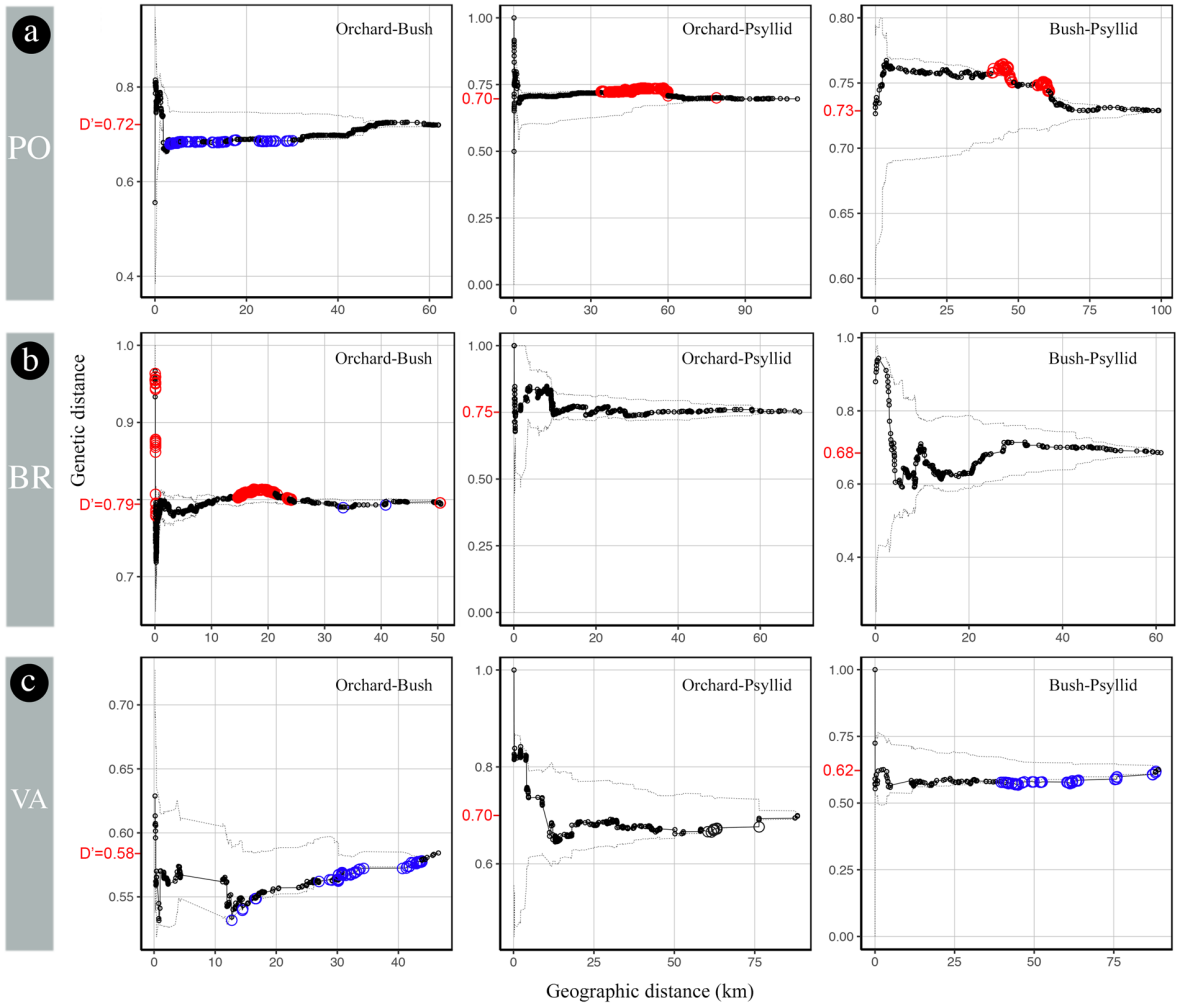
Mean pairwise genetic distances within increasing radii computed between all pairs of samples from different compartments in the (**a**) Pyrénées-Orientales, (**b**) Bas-Rhône, and (**c**) Valence regions. Solid line, observed values; dashed lines, 95% confidence envelope under the null hypothesis of spatial independence. Bigger circles below (resp., above) the envelope indicate radii within which the mean pairwise genetic distance is significantly lower (resp., higher) than expected in the absence of spatial dependence between compartments. The marginally significant mid-distance genetic proximity between orchards and bushes in the PO region is an artefact of the permutation test caused by the highly significant genetic proximity of bush samples within 30 km (Fig. 5). The values of diversity index D’ are in red on the *y*-axis. Graphs were generated using the statistical software R 3.4.0^53^, and the figure was finalized with Photoshop CS6 (https://www.adobe.com/products/photoshop.html).

For the PO region (Figs. 5a and 6a), the tests indicated a strong spatial structure (i.e., a statistically highly significant excess of genetic similarity) up to 45–50 km for both the orchard and the bush samples, but not for the psyllid samples. The diversity indices D and D’ indicate that genotypes are not genetically closer within than between compartments.

For BR, the significant genetic proximity extended only up to 8 km for the orchard samples and 200 m for the bush samples, and the psyllid samples were genetically closer up to 9 km (Figs. 5b and 6b). The diversity indices D’ for the three pairs of compartments varied in the range 0.68–0.79. This latter value for all orchard-bush pairs of samples is higher than the diversity indexes D within these two compartments (0.47 and 0.68), confirming the lack of genetic proximity between orchards and bushes.

For VA, the significant genetic proximity extended up to 35 km for the orchards, only up to 12 km for the bush samples, and the psyllid samples were genetically closer within 4 km (Figs. 5c and 6c). The diversity indices D’ for the three pairs of compartments varied in the range 0.58–0.70. This latter value for all orchard-psyllid pairs of samples is higher than the diversity indices D within these two compartments (0.56 and 0.64), confirming the overall genetic differentiation between samples from orchards and psyllids.

A striking common feature across all three regions is the absence of significant genetic proximity between different nearby compartments (Fig. 6a), which suggests limited spread at short spatio-temporal scales.

## Discussion

In this study, we used complementary statistical approaches that combine genetic data and geographical distances to identify spatial genetic patterns that provide insights into various scenarios regarding the eco-epidemiology of ‘*Ca*. P. prunorum’, including the role of humans and vectors and the associated scales of pathogen spread.

Several results of our study strengthen previous information on the functioning of the pathosystem. Firstly, we show that the pathogen is highly prevalent in all of the 61 tested bushes, confirming previous reports from other European countries^32,34^. Secondly, our study provides good estimates of the phytoplasma prevalence among French populations of immigrant psyllids (3.5–6.4%; Table 1). Overall, this low infection rate is in line with those estimated in previous studies with psyllids also captured in the natural environment in spring in France, Germany, Austria and Bulgaria^33^. Much higher infection rates in psyllids were reported locally in Italy, Switzerland and Spain, but they might have been caused by specific environmental conditions (e.g., very high level of psyllid populations in blackthorn and in untreated orchards in Swiss Valais; higher host attractiveness or palatability, as seen for Japanese plum trees in Italy), or specific local psyllid populations as seen with vectors of ‘*Ca*. P. mali’^33^. Because phytoplasma prevalence is much lower in psyllids than in bushes at the regional scale, phytoplasma acquisition from blackthorn appears to be inefficient.

Our study also provides new information about the vectors of ‘*Ca*. P. prunorum’. The discovery that *C. pruni* is actually a complex of two cryptic species^37,38^ raised questions about their respective distribution area and vector competence. In the present study, we observed balanced species proportions (Supplementary Table S5) in the PO region (59% A) and in the VA region (55% A). In contrast, in the BR region, we found almost only species A (97% A), and in northern Europe species B was the only species described^33,34,39^. The PO and VA regions may thus be part of a contact zone between the two species. The apparent absence of species A in areas where ‘*Ca*. P. prunorum’ is found in wild and cultivated *Prunus*^33,34^ strongly suggests that species B is the vector of the pathogen. Moreover, the present study supports the hypothesis that species A is also a vector of ‘*Ca*. P. prunorum’: the six major genotypes were present in both psyllid species, pathogen prevalence was similar in psyllids of species A and B and, more importantly, in bushes from the three regions (including BR where species B is very rare). However, an additional study is needed to compare the transmission capacities of the two species.

No previous study has addressed the key question of the spatial scale of ESFY epidemics. In our study, we unambiguously show a sub-regional structure of the pathogen genotypes and provide new insights into the main drivers of the epidemic. In particular, we demonstrate that the genotype distribution of the pathogen in bushes differs very significantly between the three regions (Fig. 4b), which might imply that there are few or no natural connections (i.e., psyllid dispersal) between the three major French regions where stone fruit trees are cultivated. One of the crucial unknowns in the different scenarios illustrated in Fig. 1 and Supplementary Fig. S1 was the scale of vector dispersal between pathogen acquisition and inoculation. Our study shows that pathogen genotypes are more similar than expected by chance (i.e., sites are epidemiologically connected) within a radius of 50 km or less (Fig. 5), which explains the observed strong genetic structure between the regions, which are >100 km apart. The most important result is arguably the absence of spatial dependence between the genotypes sampled from bushes and orchards within each of the three regions despite (i) the strong spatial structure of the genotypes within each compartment, and (ii) the high statistical power to detect this dependence. This means that the main drivers of the epidemic in the regions studied are neither the direct transmission of pathogens between bushes and orchards by psyllids (scenario 2) nor the successive inoculations by infectious vectors that would land in bushes and then in surrounding orchards (scenario 6). In the same vein, this also rejects scenario 14 (i.e., bounces of infectious psyllids resulting in successive inoculations to orchards and nearby blackthorns; see Supplementary Fig. 1). However, this result is unexpected because this scenario seemed compatible with the behaviour of the insect (i.e., insects flying in early spring, landing randomly and passing from one plant to another to find food, partners and egg-laying sites). The absence of spatial dependence between the genotypes sampled from bushes and orchards also excludes a local or philopatric version of scenario 4 in which bushes and neighbouring orchards would be connected through shelter sites. The presence of all major genotypes in all compartments (Fig. 4b) and the similarities in genotype distributions between compartments at the regional scale (Figs. 3 and 4a) — particularly for the PO region where more samples were collected (Table 1) — implies regional gene flows between wild and cultivated *Prunus*, i.e., scenario 4 is necessary to explain this observed pattern. However, this scenario is not sufficient to explain that the genotype composition of ‘*Ca*. P. prunorum’ is more similar across regions for orchards than for the wild compartments (Figs. 3 and 4). This observation suggests a significant contribution of inter-regional exchanges of infected plants (for planting) to the long-distance spread of the disease in orchards, i.e., scenario 8 is also necessary to explain this observed pattern. A logical consequence is that a more local version of this scenario (i.e., scenario 7_a+b_) must also contribute to the long spatial range of the genotype similarity between orchards within each region (Fig. 5). The observed patterns are not directly informative about scenarios 1, 3 and 5. However, both scenarios 1 and 3 are unlikely to significantly contribute to the observed patterns because they rely on pathogen acquisition in orchards. Except in organic farming, psyllids are very rarely observed in apricot orchards because of the probable combined effect of insecticides and the lower attractiveness of cultivated *Prunus* compared to wild *Prunus*. Furthermore, stone fruit trees are much less abundant at the regional scale than wild *Prunus*, and much less infected, as shown in our study. All these factors contribute to making this cultivated compartment a poor reservoir of inoculum and/or insects. Finally, successive inoculation events within orchards by infectious (mature) adults (scenario 5) are compatible with the persistent transmission mode. Indeed, successive inoculation events within either the orchard or the bush where an infectious vector first landed may explain (together with the planting of infected trees) that pathogen genotypes are spatially clustered within, but not between, compartments. To summarise, this field study supports and refines previous hypotheses derived from laboratory experiments^9^ by showing (i) that exchanges of infected plants contribute to disease spread between regions (a risk pointed out by several studies^30,39^), and (ii) that pathogen spread within a region involves acquisition (mainly from wild *Prunus*) by nymphs and immature adults, a first migration before the summer to one of the regional shelter hubs (conifers in altitude within several tens of kilometres), a second migration after the winter and (possibly multiple) inoculations within *Prunus* orchards after an effective latency of eight months. ESFY may therefore be seen as a self-sustaining natural pathosystem that accidentally impacts stone fruit species, particularly in apricot orchards where ESFY is essentially a monocyclic disease (i.e., with no secondary spread within the cultivated compartment). Such an epidemic cycle is an extremely unusual feature for plant diseases and evokes vector-borne zoonoses like Lyme disease or West Nile fever^40^.

These new insights into the pathosystem fill some gaps identified in an EFSA pest risk assessment^41^, including the origin of contaminations in European orchards. Indeed, this study highlights the prominent role of wild *Prunus* as an indirect source of infection for apricot trees. Given the endemic nature of this disease in Europe in wild habitats^32^ and the wide distribution area of the psyllid vectors^42^, there is little doubt that the disease cycle is similar in most diseased areas. Nevertheless, our study does not rule out the possibility that attractive *Prunus* orchards could be a significant source of inoculum in some particular situations (e.g., abandoned or organic orchards heavily infested by psyllids and the pathogen). Given the current state of knowledge about the pathosystem, once the disease is established in the natural environment, it would be unrealistic to consider eradication at any scale (farm, production basin, country). Disease control should thus focus on the protection of orchards against the psyllid vectors during the key period of their return migration after overwintering, and on the production of healthy plants for planting (in particular, by the protection of nurseries).

The approach used in this study could be applied to other pathosystems, especially in the case of plant diseases due to vector-borne bacteria (e.g., ‘*Candidatus* Phytoplasma spp.’, ‘*Candidatus* Liberibacter spp.’, *Xylella fastidiosa*), for which disease management strategies would strongly benefit from insights into the epidemiological role of wild plants or the scale of disease dispersal^6,8,10,33,43,44^. The top-down exploratory approach that we applied here can be relatively easily implemented using a single molecular marker for a clonal organism sampled from wild and cultivated compartments and a robust multi-scale statistical approach involving correspondence and join-count analyses. Further insights may be gained on the complex interaction network among host plants and insect vectors by the use of landscape genetic approaches^23,45,46^ to analyse the genotypes of ‘*Ca*. P. prunorum’ from insects collected in and at various distances from shelter sites.

## Methods

### Study area and sampling

In each growing area, we mainly sampled (90%) apricot (*Prunus armeniaca* L.) orchards (Supplementary Table S1); the remaining 10% of sampled orchards consisted of cultivated myrobalan plum (*Prunus cerasifera* Ehrh.), European plum (*Prunus domestica* L.), Japanese plum (*Prunus salicina* Lindl.) and peach (*Prunus persica* (L.) Batsch) trees. Most of the samples were collected from symptomatic trees during the autumn and winter of 2010 and 2011 (Supplementary Table S1). We also included samples obtained in previous years (2007, 2008 and 2009). From each tree, we sampled 2–3 lignified shoots from different main branches. After molecular tests to assess the presence of the phytoplasma (see below), four to six plots were chosen in each region for a more comprehensive sampling (Supplementary Table S1). This was done from winter 2010 to early spring 2011. In this second sampling, all the symptomatic trees were sampled (thus, some trees were sampled several times for confirmation of previous molecular analyses; see below). To estimate the number of asymptomatic trees (data unknown at the beginning of the study but crucial to estimate the potential role of orchards as inoculum reservoirs), one out of every three trees was also sampled (i.e., systematic sampling). Between 78 and 244 trees per plot were analysed, depending on the size of the plots (Supplementary Table S1). A total of 2,656 samples collected in 69 different orchards were used in the study (Supplementary Table S1). In parallel, we sampled wild blackthorn (*Prunus spinosa* L.) or myrobalan bushes around the plots, at up to 30–40 km (Supplementary Figs. S2, S3 and S4). We collected between 5 and 46 branches in 61 bushes (21 branches per bush, on average), for a total of 1,114 samples (Table 1; Supplementary Table S1). We massively collected mature *C. pruni* adults using a beating tray (80 × 80 cm). Other congeneric species where sometimes caught but *C. pruni* individuals were easily recognized by the colour of the forewing, which is dark brown at the apex and brown in the remaining part. Soon after identification, we conserved the samples in 96% ethanol until DNA extraction. Phytoplasma-carrying insects collected several years before in the three regions were also included in the analysis (Supplementary Table S1). Thus, a total of 2,572 psyllids sampled from 71 different bushes were analysed (Table 1; Supplementary Table S1). We recorded the GPS coordinates of all collected samples, except for the systematically sampled orchards where we attributed a unique GPS coordinate — corresponding to the centre of each plot — to all the corresponding samples (Supplementary Figs. S2, S3 and S4).

### Genetic analyses

The protocol used for the total DNA extraction from plant samples was adapted from Ahrens and Seemüller^47^. Briefly, for each plant sample, the phloem was isolated by removing the outer bark with a knife and by scraping off the layer of vascular tissue with a scalpel. Fresh phloem tissue was then ground in individual bags (0.5 g per bag). All the *Prunus* samples were individually analysed. DNA from plant samples was then purified using the CTAB method^48^ in 1.5-ml tubes. DNA pellets were diluted in 100 µl of pure water. Total DNA of individual psyllids was purified from whole bodies, and each psyllid DNA sample was assigned to species A or B by amplifying the Internal Transcribed Spacer 2 (ITS2), as previously described^37^. No individual showed two bands, demonstrating that the sample was devoid of hybrids or contamination between species.

To select samples for sequencing, ‘*Ca*. P. prunorum’ was detected in the insect and plant samples by using the ESFYf/r primers in a specific and sensitive PCR-based method, as described in Yvon *et al*.^49^. We then attempted to sequence the 1,328 positive samples at the immunodominant membrane protein (*imp*) gene locus, which was shown to be highly variable for ‘*Ca*. P. prunorum’^35^ and assumed to be present at a single copy per genome based on the known genome sequence of ‘*Ca*. P. mali’^50^, a closely related phytoplasma (i.e., belonging to the same taxonomic group, 16SrX). DNA amplification performed well for almost all of the psyllid samples, but failed for more than a quarter of the plant samples (Table 1), which we attributed to the presence of putative inhibitors like polyphenols^51^ in the unevenly infected woody material. Successfully amplified *imp* DNA was purified and Sanger-sequenced in both directions by Genewiz (Takeley, UK). Chromatograms were trimmed, assembled, and aligned using the Muscle algorithm, and visually checked under Geneious (version 5.5) (http://www.geneious.com). Sequences were deposited in GenBank (accession n° MN116709 to MN116718; Supplementary Table S6). SNPs between individual sequences were detected, and ‘*Ca*. P. prunorum’ genotypes were defined according to Danet *et al*.^35^ When previously undescribed SNPs were detected, we performed a second independent extraction from the same sample, followed by amplification and sequencing to ascertain the new *imp* sequence. To represent genealogical relationships among sequences, we used POPART software^52^ (v.1.7) to build a genotype network using the integer neighbour-joining (IntNJ) algorithm, which is well adapted for low-divergence datasets. Each infected individual contained a single *imp* genotype, except for six cultivated trees in which two different genotypes were found (either from different branches or in different years) and kept for the analysis.

### Statistical analysis

All statistical analyses were performed using R 3.4.0^53^. A correspondence analysis was performed on the contingency table (Supplementary Table S3) using the *coa* function in the *ade4* package^54^, and we visualised the results with the *factoextra* package^55^. The contingency table was also directly visualised using the *table.value* function in *ade4* to uncover specific association patterns. To test whether the distribution of genotypes among compartments differed between the three regions, we carried out multinomial regressions with the *nnet* package, and we tested the interaction between compartments and regions by comparing (using a likelihood ratio test) the complete model (including the main effects and their interaction) with the model without the interaction. Fisher’s exact tests with simulated p-value (based on 10^8^ replicates) were used to test the homogeneity of the distributions of genotypes between compartments (within each region), and of genotypes between regions (within each compartment).

In order to assess whether the sampled genotypes were spatially structured within and between compartments, the relationship between genetic and geographical distances was tested at all distances using a geostatistical method based on join counts^24^ and permutation tests. This approach is well adapted to an exploratory spatial analysis of epidemiological data because no assumption or prior knowledge of the processes of disease spread is needed (e.g., relative importance of several transmission pathways, distance and direction of spread, definition of population units)^56,57^. The genetic distance between two samples was fixed at 0 if the samples had the same genotype, and at 1 if their genotypes differed. This definition corresponds to the join count between elements of different classes of nominal data^24^. In cases where two different genotypes were detected in the same tree, their geographical distance was set at 0 (and their genetic distance at 1). In the case of a single compartment with *n* samples, the geographical distances between the *n*(*n* − 1)/2 pairs of samples were first calculated. Then, for each calculated distance *d*, we computed the average genetic distance *D*_*d*_ between the *k*_*d*_ pairs of samples separated by a geographical distance less than or equal to *d* (i.e., within a radius *d*). The average genetic distance was defined as:

$${D}_{d}=\frac{{h}_{d}}{{k}_{d}},$$

where *h*_*d*_ is the number of pairs of samples with different genotypes among the *k*_*d*_ pairs. The confidence intervals (here, at level *α* = 0.05) were obtained from *N* (here, *N* = 10,000) random permutations of the genotypes of the *n* samples. We calculated the *N* average genetic distances within each distance *d*, and the lower (respectively, upper) limit was defined as the genetic distance with rank *Nα*/2 (resp., *N*(1 − *α*/2)) among the *N* random genetic distances. A significant reduction in genetic diversity at the beginning of the curve (i.e., for the smaller radii) is expected when genotypes are spatially clustered, and the distance at which this reduced genetic diversity becomes non-significant indicates the spatial extent of genetic similarity among samples. Significant genetic distances are interpreted as false positives when non-significant genetic distances are observed at shorter geographical distances (except when these shorter distances are associated with a very low statistical power, i.e., wide confidence envelopes). Considering the cumulative number of pairs separated by a distance less than or equal to *d*, rather than the number of pairs falling in distance classes defined by intervals, provides more powerful tests and more stable curves.

When *d* is equal to the maximum distance *d*_*max*_ between two samples, we have:

$${k}_{{d}_{max}}=\frac{\,n(n-1)}{2}$$

and

$${h}_{{d}_{max}}=\frac{n(n-1)}{2}-\frac{\varSigma \,{n}_{i}({n}_{i}-1)}{2},$$

where *n*_*i*_ is the number of samples with genotype *i* and the summation is over all the genotypes. Thus, the value of ${D}_{{d}_{max}}$ is:

$$D=1-\frac{\sum {n}_{i}({n}_{i}-1)}{n(n-1)},$$

which is Simpson’s diversity index. *D* = 0 if all the samples have the same genotype, and *D* = 1 if all the samples have different genotypes. Since ${h}_{{d}_{max}}$ does not change when the genotypes are permuted, the lower and upper limits of the confidence interval are equal to *D* when *d* = *d*_*max*_. When *d* < *d*_*max*_, *D*_*d*_ can be considered as Simpson’s diversity index restricted to the pairs of samples separated by a geographical distance less than or equal to *d*.

In the case of two different compartments with *n*_1_ and *n*_2_ samples, a similar procedure was applied to the *n*_1_*n*_2_ pairs consisting of one sample of each compartment with the statistics:

$${D{\prime} }_{d}=\frac{{h{\prime} }_{d}}{{k{\prime} }_{d}},$$

where ${h{\prime} }_{d}$ and ${k{\prime} }_{d}$ have the same definition as *h*_*d*_ and *k*_*d*_, except that the two samples belong to two different compartments. However, for the computation of the confidence intervals, only the genotypes of the less structured compartment were randomly permuted to prevent false positive tests caused only by breaking the structure of the most structured compartment by permutation^58^. When the less structured compartment was also significantly structured, the tests were interpreted conservatively, i.e., interpreting the points bordering the limits of the confidence envelope as not being statistically significant. When *d* = *d*_*max*_, the value of ${D{\prime} }_{{d}_{max}}$ is:

$$D{\prime} =1-\frac{\sum {n}_{1i}{n}_{2i}}{{n}_{1}{n}_{2}},$$

where *n*_1*i*_ and *n*_2*i*_ are the numbers of samples with genotype *i* in each of the two groups. *D*′ = 0 if the two groups have the same unique genotype, and *D*’ = 1 if the two groups have no common genotype.

## Supplementary information

Supplementary information.

## Data Availability

We confirm that the Data supporting the results will be deposited in an appropriate public repository (https://data.inrae.fr).
