# Supplementary material for: Multi-scale spatial genetic structure of the vector-borne pathogen ‘Candidatus Phytoplasma prunorum’ in orchards and in wild habitats
Source: Sci Rep. 2020 Mar 19;10:5002. doi: 10.1038/s41598-020-61908-0 (PMC7081303; doi:10.1038/s41598-020-61908-0)
Supplement: Supplementary file 1 — Supplementary information. [file 41598_2020_61908_MOESM1_ESM.pdf]

Supplementary Information

Multi-scale spatial genetic structure of a the vector-borne plant pathogen '*Candidatus* phytoplasma prunorum' in orchards and in wild habitats

Véronique Marie-Jeanne[1,†], François Bonnot[1], Gaël Thébaud[1], Jean Peccoud[1,2], Gérard Labonne[1], Nicolas Sauvion[1, †, *]

[1] BGPI, Univ. Montpellier, INRAE, CIRAD, Montpellier SupAgro, Montpellier, France; [2] Current address: Université de Poitiers, Laboratoire Ecologie et Biologie des Interactions, (EBI—Joint Research Unit 7267, CNRS), 86000 Poitiers, France; † These authors equally contributed to the manuscript; * corresponding author, e-mail: nicolas.sauvion@inra.fr

Figure S1. Complete sets of eco-epidemiological scenarios illustrating the pathways through which '*Ca*. P. prunorum' might be spread in orchards and in wild habitats. Bi: bushes (i.e., wild *Prunus* = host plants); Ci: conifers (i.e., shelter plants); Ni: nurseries; Oi: orchards. In red: infected plants or infectious psyllids; in green: non-infected plants. Contamination of cultivated trees at varying spatio-temporal scales can be imagined according to the scenarios: contamination of an apricot tree by a psyllid from a nearby tree (S1) or a nearby bush (S2); contamination by an immigrant that has acquired phytoplasma on an infested tree (S3) or in a bush (S4) the previous year; multiple contaminations by the same infectious psyllid of trees from the same orchard (S5), or from a bush and then a tree (or trees) from a nearby orchard (S6); contamination of orchards by plants from nearby nurseries (S7a, S7b); contamination of different orchards by plants from the same nursery but not necessarily from the same geographic area (S8). The contamination of wild *Prunus* can be illustrated by scenarios identical to those of apricot trees (scenarios S9 to S14). Figure was generated with Photoshop CS6 (https://www.adobe.com/products/photoshop.html).

S2 Figure. Maps of the spatial distribution of samples for each compartment (bush, psyllid and orchard) surveyed in the three growing regions of the study. n: number of *imp* sequences successfully genotyped in each compartment. The maps were generated using the statistical software R 3.4.0[53] and the shapefiles of European countries from https://gadm.org/.

S3 Figure. Detailed map of the spatial distribution of samples from each compartment (bush, psyllid and orchard) surveyed in the Pyrénées-Orientales (PO) region. n: number of *imp*

sequences successfully genotyped in each compartment. A small random noise was added to the sample coordinates to avoid overlapping. The maps were generated using the statistical software R 3.4.0[53] and the shapefiles of European countries from https://gadm.org/.

S4 Figure. Detailed map of the spatial distribution of samples from each compartment (bush, psyllid and orchard) surveyed in the Bas-Rhône (BR) region. n: number of *imp* sequences successfully genotyped in each compartment. A small random noise was added to the sample coordinates to avoid overlapping. The maps were generated using the statistical software R 3.4.0[53] and the shapefiles of European countries from https://gadm.org/.

S5 Figure. Detailed map of the spatial distribution of samples from each compartment (bush, psyllid and orchard) surveyed in the Valence (VA) region. n: number of *imp* sequences successfully genotyped in each compartment. A small random noise was added to the sample coordinates to avoid overlapping. The maps were generated using the statistical software R 3.4.0[53] and the shapefiles of European countries from https://gadm.org/.

S1 Table. Summarised data for all samples surveyed in the study, ordered by region (PO, BR VA) and ecological compartment (bush, psyllid, orchard). For each compartment in each region, statistics (total number, minimum/maximum/mean values) are given for the variables: N: total number of samples collected in each region; #ESFY+: total number of samples found to be positive by PCR; %ESFY+: mean percentage of samples found to be positive by PCR; #IMP: number of samples successfully genotyped for the *imp* gene. A more concise summary of these data is provided in Table 1.

S2 Table. Contingency table of the frequency distribution of all *imp* genotypes successfully sequenced in each ecological compartment (bush, psyllid and orchard) and each of the three growing regions. Genotypes never described in previous studies are italicised. The legend in Fig. 2 explains how these new genotypes were named. Asterisks indicate changes in the amino acid sequence.

S3 Table. Contingency table displaying the frequency distribution of the six major *imp* genotypes in each ecological compartment (bush, psyllid and orchard) and each of the three growing regions of the study. PO: Pyrénées-Orientales; BR: Bas-Rhône; VA: Valence. Very

rare genotypes (i.e., with frequency < 1%, combining all regions) were excluded from the analyses. See Supplementary Table S2 for more details.

S4 Table. Relative proportions of the six major *imp* genotypes in the three ecological compartments and the three regions. PO: Pyrénées-Orientales; BR: Bas-Rhône; VA: Valence. This table is summarised in Fig. 4.

S5 Table. Number of major *imp* genotypes sequenced for each species of the *Cacopsylla pruni* complex, all regions combined and region-by-region. PO: Pyrénées-Orientales; BR: Bas-Rhône; VA: Valence. #A/B: total number of psyllids collected from species A and/or B. #ESFY+: total number of samples found to be positive by PCR. %A/B: proportion of psyllids of each psyllid species. %ESFY+: proportion of each psyllid species among the PCR-positive psyllids.

S6 Table. List of the *imp* genotypes described to date, with their GenBank accession numbers.

S1 Figure. Complete sets of eco-epidemiological scenarios illustrating the pathways through which '*Ca.* P. prunorum' might be spread in orchards and wild habitat

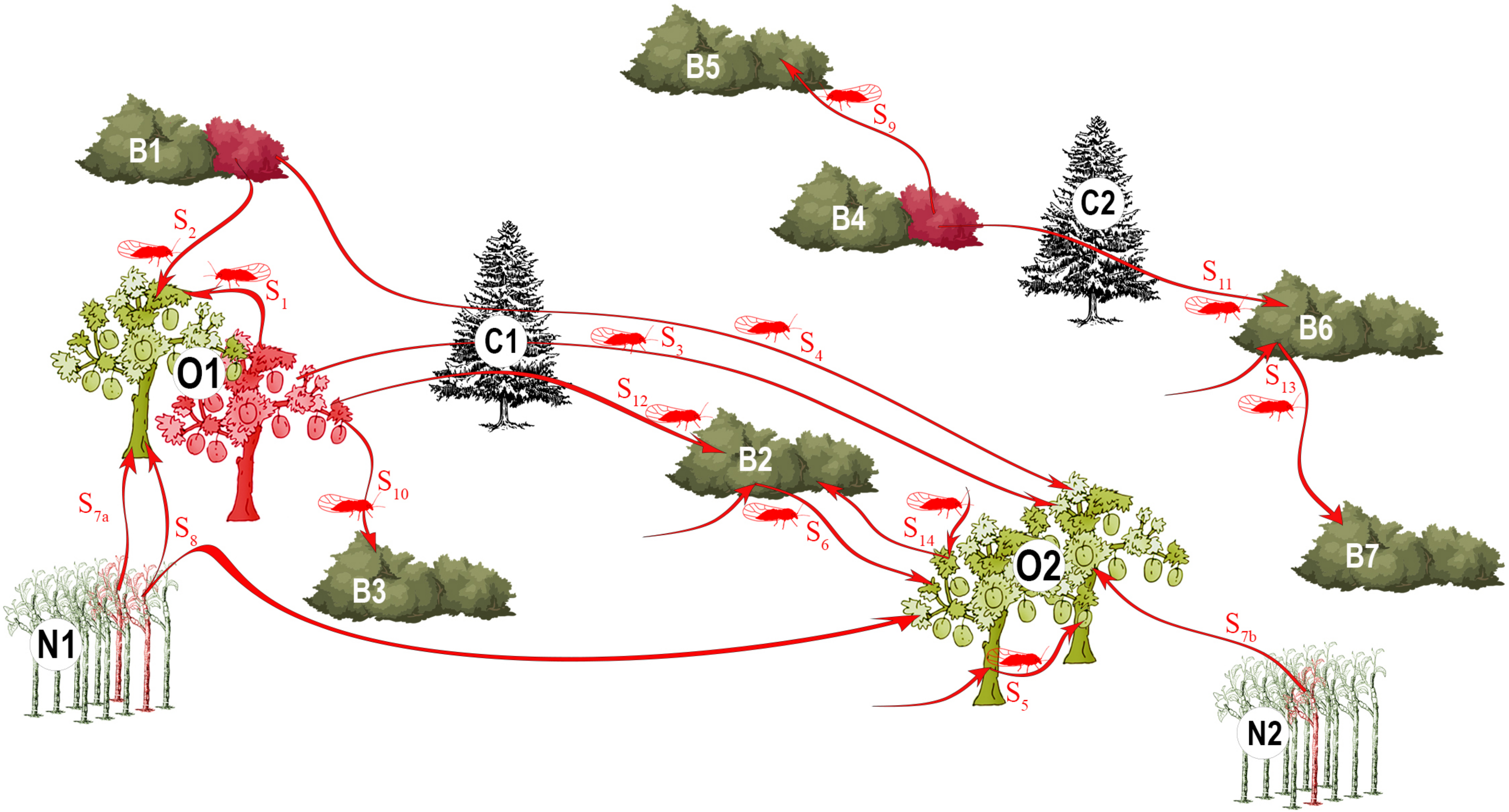

Figure S2. Maps of the spatial distribution of samples for each compartment (bush, psyllid, and orchard) surveyed in the three growing regions of the study.

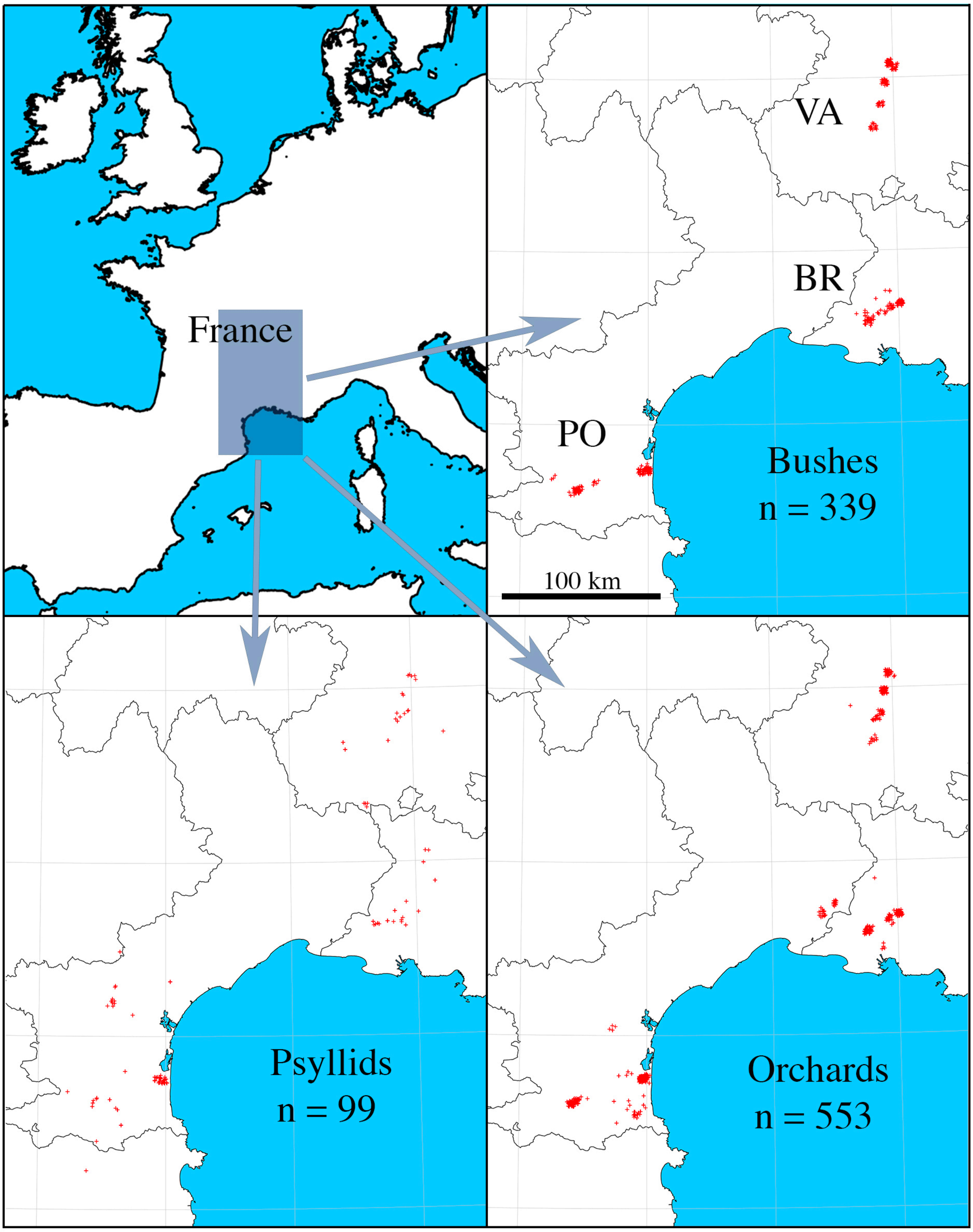

Figure S3. Detailed map of the spatial distribution of samples from each compartment (bush, psyllid, and orchard) surveyed in the Pyrénées-Orientales (PO) region.

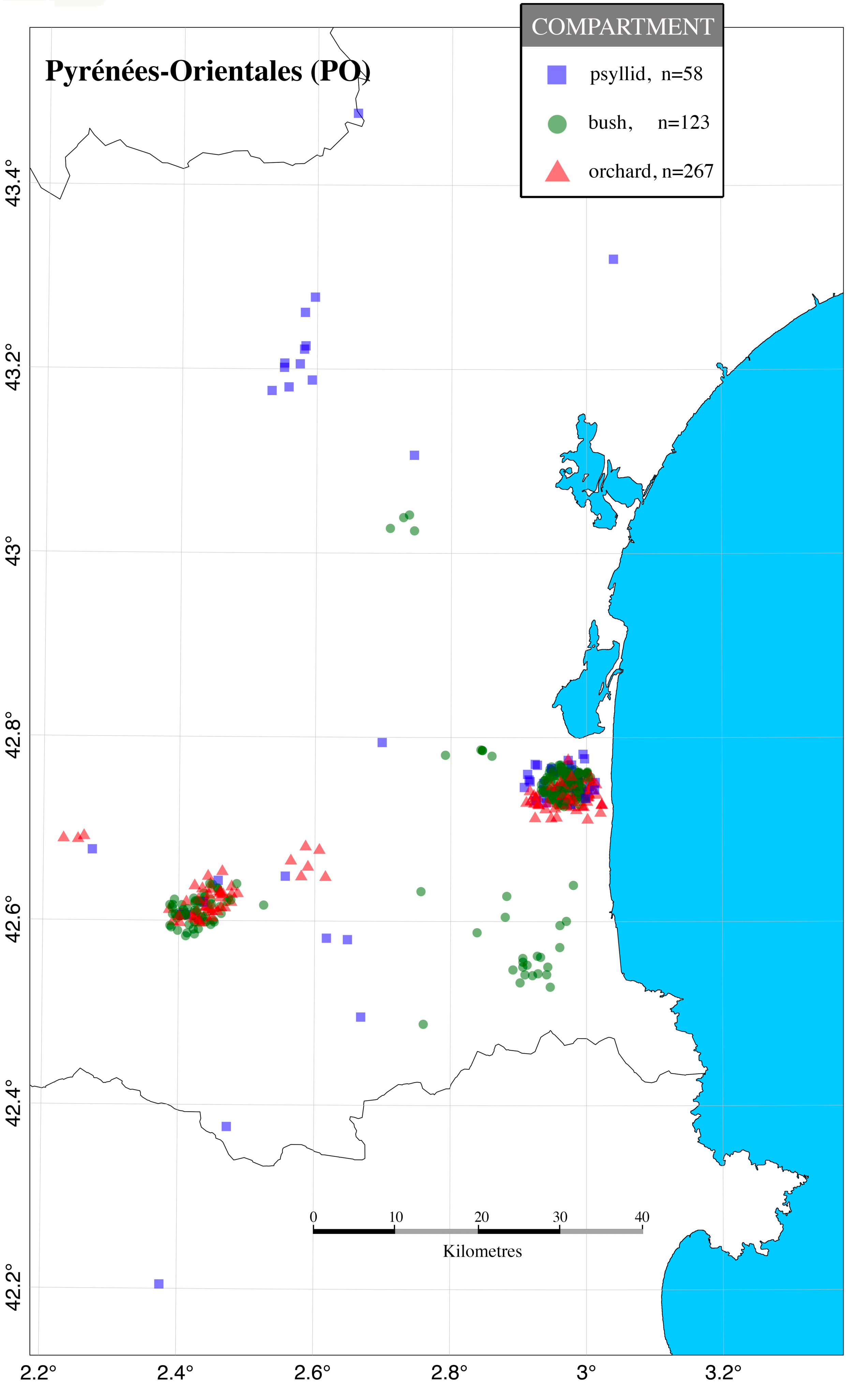

Figure S4. Detailed map of the spatial distribution of samples from each compartment (bush, psyllid, and orchard) surveyed in the Bas-Rhône (BR) region.

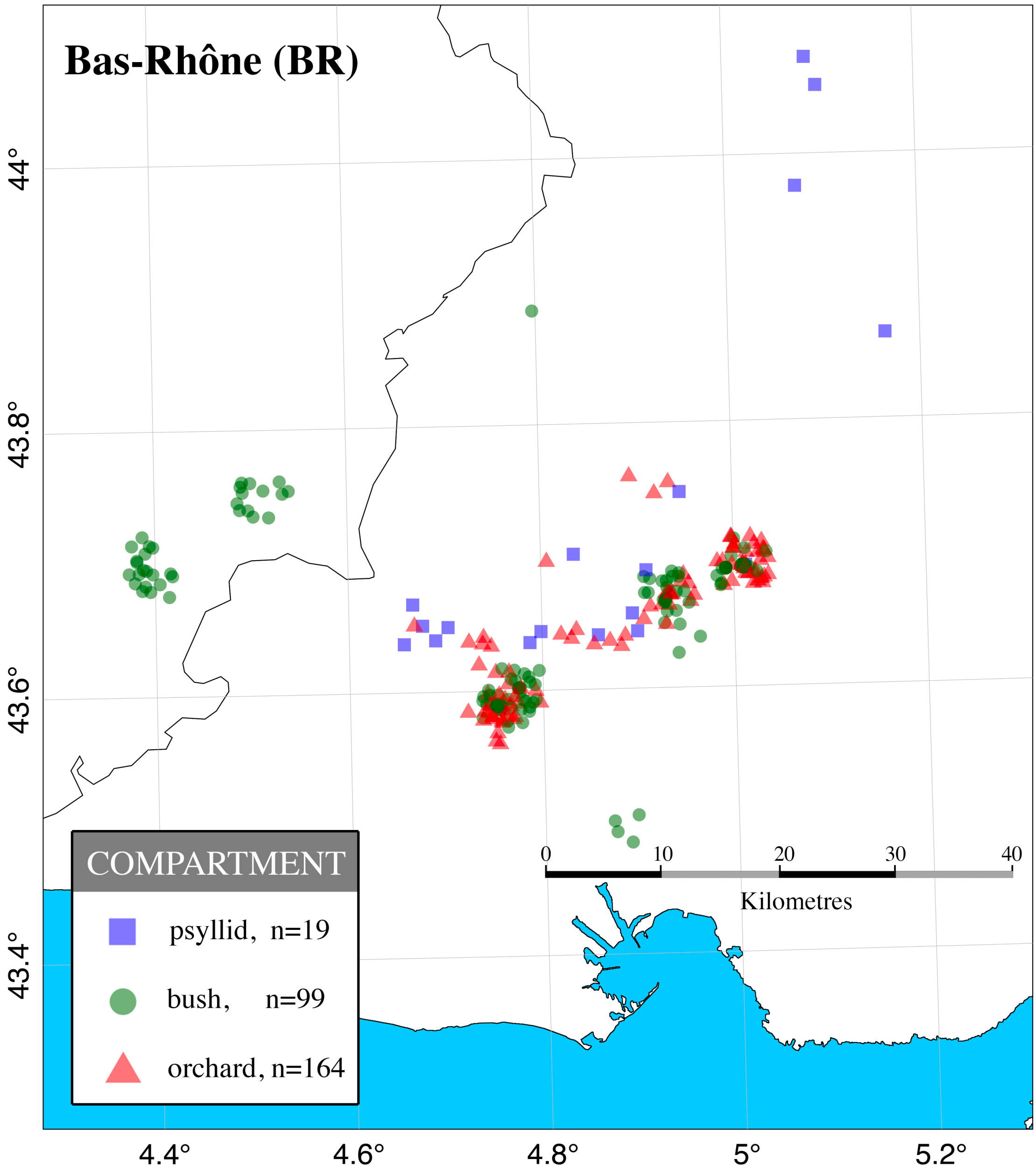

Figure S5. Detailed map of the spatial distribution of samples from each compartment (bush, psyllid, and orchard) surveyed in the Valence (VA) region.

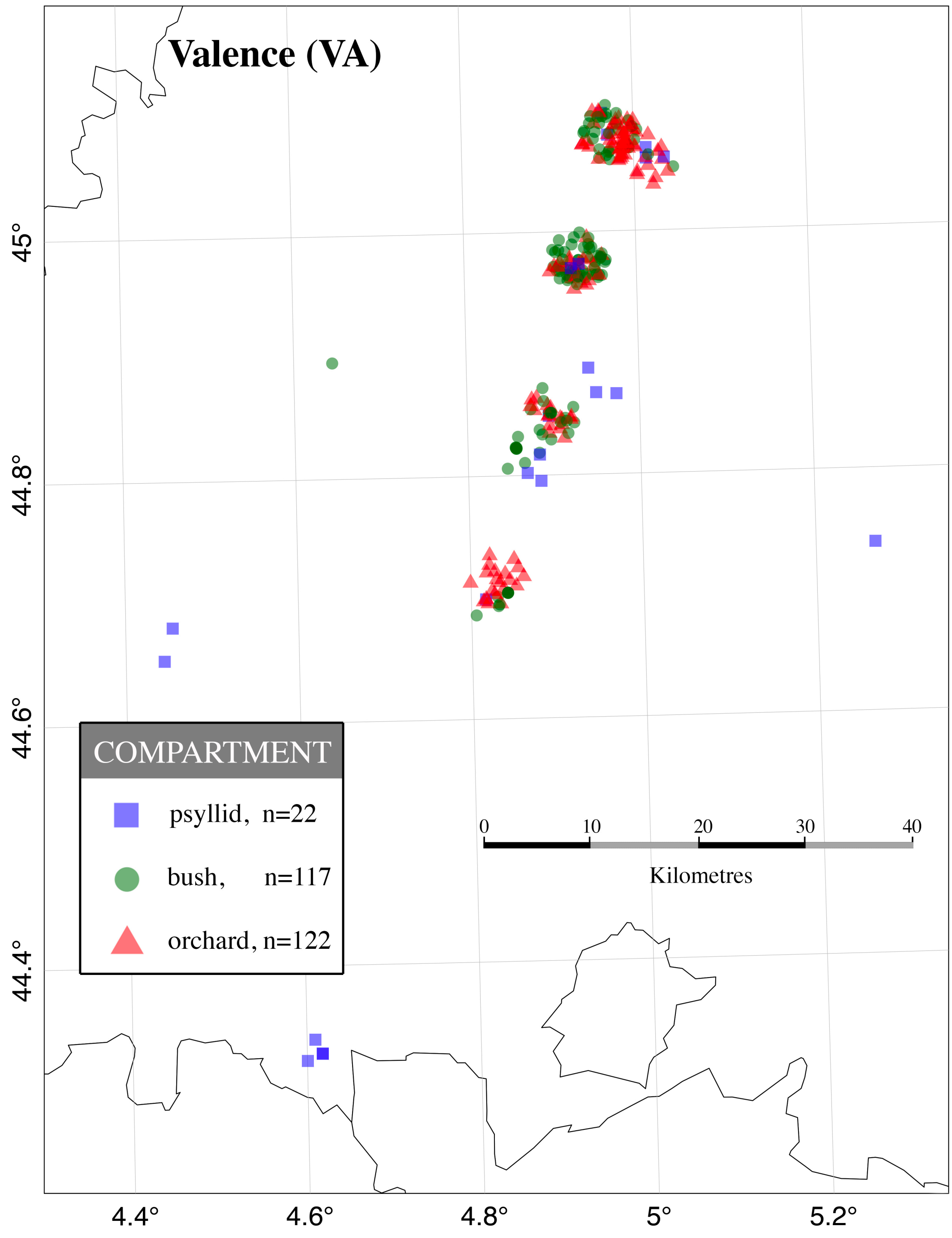

Supplementary Table S1. Summarised data for all samples surveyed in the study, ordered by region (PO, BR, VA) and ecological compartment (bush, psyllid, orchard).

Region	Compartment	Host plant	Sub-region	Locality	Bush codename	N			#ESFY+	%ESFY+	#IMP	Sampling date
PO	bush	wild Prunus	Prades	Bouleternère	M01	37			8	21.62	6	01/04/2010
PO	bush	wild Prunus	Prades	Eus	M02	30			12	40.00	11	01/04/2010
PO	bush	wild Prunus	Prades	Prades	M03	46			36	78.26	25	01/04/2010
PO	bush	wild Prunus	Prades	Sirach	M04	12			3	25.00	3	01/04/2010
PO	bush	wild Prunus	Prades	Codalet	M05	16			8	50.00	5	01/04/2010
PO	bush	wild Prunus	Torreilles	St Laurent de la Salanque	M07	26			4	15.38	1	02/04/2010
PO	bush	wild Prunus	Torreilles	Bompas	M08	32			30	93.75	21	01/04/2010
PO	bush	wild Prunus	Torreilles	Claira	M09	25			0	0.00	0	09/12/2010
PO	bush	wild Prunus	Torreilles	Sica Centrex	M10	30			2	6.67	1	28/04/2011
PO	bush	wild Prunus	Torreilles	Sainte-Marie	M11	22			22	100.00	18	01/04/2010
PO	bush	wild Prunus	Torreilles	Torreilles	M12	26			24	92.31	19	01/04/2010
PO	bush	wild Prunus	Torreilles	Torreilles	M13	10			10	100.00	9	01/04/2010
PO	bush	wild Prunus	Prades	Col de Jau	M90	10			3	30.00	3	12/10/2011
PO	bush	wild Prunus	Prades	Villerach	M91	10			1	10.00	1	10/03/2008
				STATISTICS	N	14			14	14	14	
					∑	332			163		123	
					min	10			0	0.00	0	
					max	46			36	100.00	25	
					mean	23.71			11.8	47.36	9.13	
					SE	2.96			3.12	10.09	2.29	
**Region**	**Compartment**	**Host plant**	**Sub-region**	**Locality**	**Psyllid codename**	**N sp.A+B**	**N sp.A**	**N sp.B**	**#ESFY+**	**%ESFY+**	**#IMP**	**Sampling date**
PO	psyllid	wild Prunus	Autre	Valfogona	E025	25	25	0	1	4.00	1	23/04/2005
PO	psyllid	wild Prunus	Prades	Col de Jau	E049	24	0	24	1	4.17	1	21/11/2005
PO	psyllid	wild Prunus	Prades	Eus	E051	27	27	0	1	3.70	1	02/03/2006
PO	psyllid	wild Prunus	Torreilles	Torreilles	E102	49	29	20	2	4.08	2	../04/2006
PO	psyllid	wild Prunus	Torreilles	Torreilles	E203	77	35	42	5	6.49	3	25/02/2007
PO	psyllid	wild Prunus	Torreilles	Torreilles	E204	90	45	45	5	5.56	4	04/03/2007
PO	psyllid	wild Prunus	Torreilles	Torreilles	E205	54	22	32	1	1.82	1	11/03/2007
PO	psyllid	wild Prunus	Torreilles	Torreilles	E206	30	13	17	2	6.67	2	18/03/2007
PO	psyllid	wild Prunus	Torreilles	Torreilles	E208	34	9	25	4	11.76	4	02/04/2007
PO	psyllid	wild Prunus	Torreilles	Torreilles	E212	21	4	17	1	4.55	1	23/05/2007
PO	psyllid	wild Prunus	Torreilles	Torreilles	E214	7	7	0	1	14.28	1	31/01/2007
PO	psyllid	wild Prunus	Torreilles	Torreilles	E216	84	48	36	3	3.53	3	21/02/2007
PO	psyllid	wild Prunus	Torreilles	Torreilles	E217	63	28	35	2	3.17	2	28/02/2007
PO	psyllid	wild Prunus	Torreilles	Torreilles	E218	108	31	77	2	1.80	2	07/03/2007
PO	psyllid	wild Prunus	Torreilles	Torreilles	E219	40	13	27	1	2.50	1	14/03/2007
PO	psyllid	wild Prunus	Torreilles	Torreilles	E220	49	15	34	1	2.04	1	28/03/2007
PO	psyllid	wild Prunus	Autre	Capendu	E245	28	15	13	2	7.14	2	12/03/2008
PO	psyllid	wild Prunus	Autre	Thézan	E246	20	12	8	1	5.00	1	12/03/2008
PO	psyllid	wild Prunus	Prades	Col d'Ares	E255	26	25	1	1	3.85	1	14/03/2008
PO	psyllid	wild Prunus	Prades	Palalda	E258	30	30	0	1	3.13	1	14/03/2008
PO	psyllid	wild Prunus	Prades	Boule d'Amont	E259	48	48	0	2	4.17	2	14/03/2008
PO	psyllid	wild Prunus	Prades	La Bastide R.	E269	8	3	5	1	12.50	1	18/03/2008
PO	psyllid	wild Prunus	Prades	Prades	E560	104	104	0	2	1.92	2	02/04/2010
PO	psyllid	wild Prunus	Prades	Sirach	E561	196	189	7	1	0.67	1	02/04/2010
PO	psyllid	wild Prunus	Prades	Codalet	E562	60	58	2	1	1.49	1	02/04/2010
PO	psyllid	wild Prunus	Prades	Rodès	E563	82	72	10	1	1.06	1	02/04/2010
PO	psyllid	wild Prunus	Autre	Capestang	E566	45	6	39	1	2.04	1	06/04/2010
PO	psyllid	wild Prunus	Autre	Rieux en Minervois	E567	42	2	40	2	4.00	2	06/04/2010
PO	psyllid	wild Prunus	Torreilles	St Laurent de la Salanque	E569	72	32	40	4	5.00	4	01/04/2010
PO	psyllid	wild Prunus	Torreilles	St Laurent de la Salanque	E665	12	10	2	1	8.33	1	02/02/2011
PO	psyllid	wild Prunus	Autre	Puicheric	E744	75	13	62	6	2.61	6	28/03/2012
PO	psyllid	wild Prunus	Torreilles	Tautavel	E746	93	45	48	1	1.07	1	28/03/2012
				STATISTICS	N	32	32	32	32	32	32	
					∑	1723	1015	708	61		58	
					min	7	0	0	1	0.67	1	
					max	196	189	77	6	14.29	6	
					mean	53.84	31.72	22.13	1.91	3.54	1.81	
					SE	6.80	6.44	3.61	0.25		0.22	
**Region**	**Compartment**	**Host plant**	**Sub-region**	**Locality**	**Plot codename**	**N cumulative sampling**	**N per date**		**#ESFY+**	**%ESFY+**	**#IMP**	**Sampling date**
PO	orchard	Prunus armeniaca	Torreilles	Sica Centrex	GL01	3	3		3		3	../04/2008
PO	orchard	Prunus cerasifera	Torreilles	Sica Centrex	GL02	1	1		1		1	../04/2008
PO	orchard	Prunus armeniaca	Torreilles	Sica Centrex	GL03	1	1		1		1	../04/2008
PO	orchard	Prunus armeniaca	Torreilles	Sica Centrex	GL04	4	4		4		5	../04/2008
PO	orchard	Prunus persica	Torreilles	Sica Centrex	GL05	2	2		2		2	../04/2008
PO	orchard	Prunus armeniaca	Torreilles	Marquixanes	GL06	2	2		2		1	03/03/2009
PO	orchard	Prunus armeniaca	Torreilles	Alenya	GL07	2	2		2		1	03/03/2009
PO	orchard	Prunus armeniaca	Torreilles	Villeplaine	GL08	1	1		1		1	03/03/2009
PO	orchard	Prunus armeniaca	Torreilles	Villeneuve	GL09	1	1		1		1	03/03/2009
PO	orchard	Prunus armeniaca	Prades	Ria	P01	1	1		1		1	03/02/2010
PO	orchard	Prunus armeniaca	Prades	Sirach	P02	17	17		17		17	03/02/2010
PO	orchard	Prunus armeniaca	Prades	Codalet	P03a	3	3		3		3	03/02/2010

PO	orchard	Prunus armeniaca	Prades	Codalet	P03b	118	106		26	24.56	29	03/02/2010
PO	orchard	Prunus armeniaca	Prades	Codalet			12		5			18/03/2011
PO	orchard	Prunus armeniaca	Prades	Lloncet	P04	135	117		17	14.53	27	03/02/2010
PO	orchard	Prunus armeniaca	Prades	Lloncet			18		18			10/03/2011
PO	orchard	Prunus armeniaca	Torreilles	Sica Centrex	P05	2	2		2		2	03/02/2010
PO	orchard	Prunus armeniaca	Torreilles	Royal Sophie	P06	80	2		2		4	03/02/2010
PO	orchard	Prunus armeniaca	Torreilles	Royal Sophie			78		4	5.13		09/12/2010
PO	orchard	Prunus armeniaca	Torreilles	Claira-Annexe 2	P07	40	2		2		2	12/02/2008
PO	orchard	Prunus armeniaca	Torreilles	Claira-Annexe 2			21		21		21	../04/2009
PO	orchard	Prunus armeniaca	Torreilles	Claira-Annexe 2			3		1		0	01/04/2010
PO	orchard	Prunus armeniaca	Torreilles	Claira-Annexe 2			14		14		14	../04/2012
PO	orchard	Prunus armeniaca	Torreilles	Claira-Monilia	P08	255	40		38		81	09/12/2010
PO	orchard	Prunus armeniaca	Torreilles	Claira-Monilia			9		8			05/01/2011
PO	orchard	Prunus armeniaca	Torreilles	Claira-Monilia			206		81	39.32		28/04/2011
PO	orchard	Prunus armeniaca	Torreilles	Claira	P10	206	206		4	1.94	2	09/12/2010
PO	orchard	Prunus armeniaca	Torreilles	Ecoles	P11	4	4		4		4	03/02/2010
PO	orchard	Prunus armeniaca	Torreilles	Entrée Village	P12	10	10		10		10	03/02/2010
PO	orchard	Prunus armeniaca	Torreilles	Sainte-Marie	P13	2	2		2		1	03/02/2010
PO	orchard	Prunus armeniaca	Torreilles	Los Ecorchats	P14	3	3		3		3	03/02/2010
PO	orchard	Prunus armeniaca	Estagel-Rivesaltes	Cases de Pène	P16	1	1		1		1	03/02/2010
PO	orchard	Prunus armeniaca	Estagel-Rivesaltes	Espira de l'Agly	P17	2	2		2		2	17/02/2010
PO	orchard	Prunus armeniaca	Estagel-Rivesaltes	Espira de l'Agly	P18	1	1		1		1	17/02/2010
PO	orchard	Prunus armeniaca	Estagel-Rivesaltes	Rivesaltes	P19	1	1		1		1	17/02/2010
PO	orchard	Prunus armeniaca	Estagel-Rivesaltes	Jonquières	P20	4	4		4		4	17/02/2010
PO	orchard	Prunus armeniaca	Autre	St Genies des Fontaines	P21	7	7		7		7	../04/2009
PO	orchard	Prunus armeniaca	Autre	St Genies des Fontaines	P21	5	5		5		5	17/02/2010
PO	orchard	Prunus armeniaca	Autre	Ceret	P23	3	3		1		1	17/02/2010
PO	orchard	Prunus armeniaca	Autre	Bages	P24	2	2		2		2	17/02/2010
PO	orchard	Prunus armeniaca	Autre	Elne	P26	2	2		2		2	17/02/2010
PO	orchard	Prunus armeniaca	Torreilles	Sica Centrex	P27	32	32		5		4	17/02/2010
				STATISTICS	N	34			42	5	37	
					∑	953			331		267	
					min	1			1	1.94	0	
					max	255			81	39.32	81	
					mean	28.03			7.88	17.09	7.22	
					SE	10.45			2.16	6.81	2.37	

Region	Compartment	Host plant	Sub-region	Locality	Bush codename	N			#ESFY+	%ESFY+	#IMP	Sampling date
BR	bush	wild Prunus	La Dynamite	Arles	M50	10			2	20.00	1	24/03/2009
BR	bush	wild Prunus	La Dynamite	La Dynamite	M51	5			5	100.00	5	24/03/2009
BR	bush	wild Prunus	La Dynamite	La Dynamite	M52	20			10	50.00	8	27/03/2010
BR	bush	wild Prunus	La Dynamite	La Dynamite	M53	20			7	35.00	6	11/04/2010
BR	bush	wild Prunus	La Dynamite	La Dynamite	M54	10			9	90.00	3	16/02/2010
BR	bush	wild Prunus	La Dynamite	La Dynamite	M55	10			2	20.00	1	27/03/2010
BR	bush	wild Prunus	La Samatane	La Samatane	M56	15			1	6.67	1	24/03/2009
BR	bush	wild Prunus	La Samatane	La Samatane	M57	10			2	20.00	1	24/03/2009
BR	bush	wild Prunus	La Samatane	La Samatane	M58	10			8	80.00	4	24/03/2009
BR	bush	wild Prunus	La Samatane	La Samatane	M59	10			2	20.00	0	24/03/2009
BR	bush	wild Prunus	La Samatane	La Samatane	M60	20			3	15.00	2	27/03/2009
BR	bush	wild Prunus	La Samatane	La Samatane	M61	10			2	20.00	1	27/03/2009
BR	bush	wild Prunus	La Samatane	La Samatane	M62	10			2	20.00	1	27/03/2009
BR	bush	wild Prunus	La Samatane	La Samatane	M63	10			3	30.00	2	27/03/2009
BR	bush	wild Prunus	La Samatane	La Samatane	M64	10			1	10.00	1	27/03/2009
BR	bush	wild Prunus	La Samatane	La Samatane	M65	10			4	40.00	3	27/03/2009
BR	bush	wild Prunus	La Samatane	La Samatane	M66	10			5	50.00	5	27/03/2009
BR	bush	wild Prunus	Eyguières	Eyguières	M67	10			5	50.00	3	27/03/2009
BR	bush	wild Prunus	Eyguières	Eyguières	M68	20			8	40.00	7	27/03/2009
BR	bush	wild Prunus	Eyguières	Eyguières	M69	10			5	50.00	3	27/03/2009
BR	bush	wild Prunus	Eyguières	Eyguières	M70	10			8	80.00	5	07/02/2010
BR	bush	wild Prunus	Eyguières	Eyguières	M71	20			6	30.00	5	07/02/2010
BR	bush	wild Prunus	Eyguières	Eyguières	M71	20			6	30.00		27/03/2010
BR	bush	wild Prunus	Eyguières	Eyguières	M72	10			2	20.00	0	27/03/2010
BR	bush	wild Prunus	Eyguières	Eyguières	M73	10			9	90.00	12	07/02/2010
BR	bush	wild Prunus	Eyguières	Eyguières	M73	20			5	25.00		27/03/2010
BR	bush	wild Prunus	La Samatane	La Samatane	M74	10			2	20.00	1	27/03/2010
BR	bush	wild Prunus	Eyguières	Eygalières	M75	10			4	40.00	3	24/03/2009
BR	bush	wild Prunus	La Dynamite	La Dynamite	M76	40			9	22.50	6	27/03/2010
BR	bush	wild Prunus	La Dynamite	La Dynamite	M77	10			8	80.00	5	11/04/2010
BR	bush	wild Prunus	La Dynamite	La Dynamite	M78	6			3	50.00	1	11/04/2010
BR	bush	wild Prunus	La Dynamite	Saint Martin-de-Crau	M79	10			4	40.00	3	24/03/2009
BR	bush	wild Prunus	La Dynamite	Saint Martin-de-Crau	M80	10			0	0.00	0	24/03/2009
				STATISTICS	N	33			33	33	31	
					∑	426			152		99	
					min	5			0	0.00	0	
					max	40			10	100.00	12	
					mean	12.91			4.61	39.22	3.19	
					SE	1.15			0.49	4.58	0.49	

Region	Compartment	Host plant	Sub-region	Locality	Psyllid codename	N sp.A+B	N sp.A	N sp.B	#ESFY+	%ESFY+	#IMP	Sampling date
BR	psyllid	wild Prunus		Carpentras	E289	37	36	1	2	5.40	2	26/03/2008
BR	psyllid	wild Prunus		Aureilles	E367	10	10	0	1	10.00	1	18/03/2009
BR	psyllid	wild Prunus		Cavaillon	E371	58	58	0	2	3.45	2	18/03/2009
BR	psyllid	wild Prunus		Arles	E380	14	10	4	5	35.71	5	21/03/2009
BR	psyllid	wild Prunus		St Martin de Crau	E381	15	15	0	2	13.33	2	21/03/2009
BR	psyllid	wild Prunus		St Martin de Crau	E383	38	36	2	1	2.63	1	21/03/2009
BR	psyllid	wild Prunus		St Martin de Crau	E385	52	52	0	2	3.85	2	21/03/2009
BR	psyllid	wild Prunus		Eygalières	E386	13	13	0	1	7.70	1	21/03/2009
BR	psyllid	wild Prunus		Mouriès	E387	54	53	1	1	1.85	1	21/03/2009
BR	psyllid	wild Prunus		Peynes-les-Fontaines	E448	2	2	0	1	50.00	1	09/03/2009
BR	psyllid	wild Prunus		Eygalières	E616	6	6	0	1	16.67	1	26/03/2006
				STATISTICS	N	11	11	11	11	11	11	
					∑	299	291	8	19		19	
					min	2	2	0	1	1.85	1	
					max	58	58	4	5	50.00	5	
					mean	27.18	26.46	0.73	1.73	6.35	1.73	
					SE	6.32	6.33	0.38	0.36	4.67	0.36	
**Region**	**Compartment**	**Host plant**	**Sub-region**	**Locality**	**Plot codename**	**N cumulative sampling**	**N per date**		**#ESFY+**	**%ESFY+**	**#IMP**	**Sampling date**
BR	orchard	Prunus armeniaca	Saint-Gilles	St Gilles	P60	23	23		23		18	../04/2008
BR	orchard	Prunus persica	Saint-Gilles	Générac	P61	2	2		2		2	09/02/2009
BR	orchard	Prunus domestica	Saint-Gilles	Bellegarde	P62	24	24		24		13	../04/2008
BR	orchard	Prunus salicina	La Dynamite	La Dynamite	P63a	8	8		8		7	../04/2002
BR	orchard	Prunus armeniaca	La Dynamite	La Chapelette	P63b	139	13		13		24	16/02/2010
BR	orchard	Prunus armeniaca	La Dynamite	La Chapelette			122		20	16.39		23/10/2010
BR	orchard	Prunus armeniaca	La Dynamite	La Chapelette			4		2			26/02/2011
BR	orchard	Prunus armeniaca	La Dynamite	Aulnes	P64a	140	4		4		4	16/02/2010
BR	orchard	Prunus armeniaca	La Dynamite	Aulnes			124		14	11.29	6	21/10/2010
BR	orchard	Prunus armeniaca	La Dynamite	Aulnes			12		7		3	26/01/2011
BR	orchard	Prunus salicina	La Dynamite	Aulnes	P64b	23	23		8		8	26/02/2011
BR	orchard	Prunus armeniaca	Istres	Istres	P65	2	2		1		1	../04/2010
BR	orchard	Prunus armeniaca	Istres	Istres	P66	24	24		3		3	../04/2010
BR	orchard	Prunus armeniaca	La Samatane	La Samatane	P67	8	8		4		0	../04/2006
BR	orchard	Prunus armeniaca	La Samatane	La Samatane	P68a=D11	17	9		9		12	../04/2006
BR	orchard	Prunus armeniaca	La Samatane	La Samatane			8		8			24/03/2009
BR	orchard	Prunus armeniaca	La Samatane	La Samatane	P68b=D12	103	101		4	3.96	3	21/10/2010
BR	orchard	Prunus armeniaca	La Samatane	La Samatane			2		1			26/02/2011
BR	orchard	Prunus armeniaca	La Samatane	La Samatane	P68c=D15	118	8		8		9	24/03/2009
BR	orchard	Prunus armeniaca	La Samatane	La Samatane			110		28	25.45		21/10/2010
BR	orchard	Prunus armeniaca	La Samatane	La Samatane	P68d=E15	2	2		2		2	24/03/2009
BR	orchard	Prunus armeniaca	Eyguières	Eyguières	P69	8	8		8		8	16/02/2010
BR	orchard	Prunus armeniaca	Eyguières	Eyguières	P70	17	17		17		17	16/02/2010
BR	orchard	Prunus armeniaca	Eyguières	Eyguières	P71a	112	1		1		1	07/02/2010
BR	orchard	Prunus armeniaca	Eyguières	Eyguières			104		5	4.81	1	28/10/2010
BR	orchard	Prunus armeniaca	Eyguières	Eyguières			7		2		1	26/02/2011
BR	orchard	Prunus armeniaca	Eyguières	Eyguières	P71b	119	1		1		1	07/02/2010
BR	orchard	Prunus armeniaca	Eyguières	Eyguières			117		15	12.82	5	23/10/2010
BR	orchard	Prunus armeniaca	Eyguières	Eyguières			1		0		0	26/02/2011
BR	orchard	Prunus armeniaca	Eyguières	Eyguières	P71c	4	1		1		14	07/02/2010
BR	orchard	Prunus armeniaca	Eyguières	Eyguières			1		1			28/10/2010
BR	orchard	Prunus armeniaca	Eyguières	Eyguières			2		2			26/02/2011
BR	orchard	Prunus armeniaca	Eyguières	Eyguières	P71d	1	1		1			07/02/2010
BR	orchard	Prunus armeniaca	Eyguières	Eyguières	P71e	2	2		2			07/02/2010
BR	orchard	Prunus armeniaca	Eyguières	Eyguières	P71f	2	2		2			27/03/2010
BR	orchard	Prunus armeniaca	Eyguières	Eyguières	P71g	2	2		2			07/02/2010
BR	orchard	Prunus persica	Avignon	Avignon	P72	1	1		1		1	07/02/2010
				STATISTICS	N	24			37	6	26	
					∑	901			254		164	
					min	1			0	3.96	0	
					max	140			28	25.89	24	
					mean	37.54			6.86	12.45	6.31	
					SE	10.40			1.22	3.25	1.26	
**Region**	**Compartment**	**Host plant**	**Sub-region**	**Locality**	**Bush codename**	**N**			**#ESFY+**	**%ESFY+**	**#IMP**	**Sampling date**
VA	bush	wild Prunus	Romans	Les Balmes	M20	31			22	70.97	19	09/04/2010
VA	bush	wild Prunus	Romans	Saint-Bardoux	M22	30			16	53.33	16	08/04/2010
VA	bush	wild Prunus	Romans	Saint-Bardoux	M23	30			11	36.67	11	09/04/2010
VA	bush	wild Prunus	Romans	Saint-Bardoux	M24	33			12	36.36	10	09/04/2010
VA	bush	wild Prunus	Plaine de Valence	Beauvallon	M25	30			19	63.33	11	09/04/2010
VA	bush	wild Prunus	Plaine de Valence	Beauvallon	M26	19			11	57.89	6	09/04/2010
VA	bush	wild Prunus	Mirmande	Mirmande	M27	20			20	100.00	6	09/04/2010
VA	bush	wild Prunus	Mirmande	Mirmande	M28	27			15	55.56	13	09/04/2010
VA	bush	wild Prunus	Mirmande	Mirmande	M29	24			5	20.83	1	09/04/2010
VA	bush	wild Prunus	Plaine de Valence	Gotheron	M30	20			11	55.00	8	09/04/2010

VA	bush	wild Prunus	Plaine de Valence	Gotheron	M31	18			0	0.00	0	09/04/2010
VA	bush	wild Prunus	Plaine de Valence	Gotheron	M32	16			1	6.25	1	09/04/2010
VA	bush	wild Prunus	Plaine de Valence	Gotheron	M33	26			1	3.84	1	09/04/2010
VA	bush	wild Prunus	Plaine de Valence	Gotheron	M34	32			15	46.87	14	09/04/2010
				STATISTICS	N	14			14	14	14	
					∑	356			159		117	
					min	16			0	0.00	0	
					max	33			22	100.00	19	
					mean	25.43			11.36	43.35	8.36	
					SE	1.56			1.93	7.56	1.63	
**Region**	**Compartment**	**Host plant**	**Sub-region**	**Locality**	**Psyllid codename**	**N sp.A+B**	**N sp.A**	**N sp.B**	**#ESFY+**	**%ESFY+**	**#IMP**	**Sampling date**
VA	psyllid	wild Prunus	Mirmande	St Marcel d'Ardèche	E003	6	6	0	3	50.00	2	12/04/2005
VA	psyllid	wild Prunus	Mirmande	Vesseaux	E094	20	20	0	1	5.00	1	13/04/2006
VA	psyllid	wild Prunus	Plaine de Valence	Etoile-sur-Rhône	E117	56	50	6	3	4.80	3	15/04/2006
VA	psyllid	wild Prunus	Plaine de Valence	Etoile-sur-Rhône	E119	7	6	1	1	14.30	1	09/04/2006
VA	psyllid	wild Prunus	Mirmande	St Marcel d'Ardèche	E321	3	3	0	1	33.33	1	29/03/2007
VA	psyllid	wild Prunus	Mirmande	Vesseaux	E441	15	12	3	1	5.90	1	14/04/2009
VA	psyllid	wild Prunus	Romans	Saint-Bardoux	E580	32	7	25	1	2.90	1	08/04/2010
VA	psyllid	wild Prunus	Romans	Saint-Bardoux	E581	32	10	22	1	3.26	1	08/04/2010
VA	psyllid	wild Prunus	Romans	Saint-Bardoux	E582	31	4	27	1	3.03	1	09/04/2010
VA	psyllid	wild Prunus	Romans	Saint-Bardoux	E583	31	9	22	0	0.00		09/04/2010
VA	psyllid	wild Prunus	Romans	Peyrins	E584	8	2	6	0	0.00		09/04/2010
VA	psyllid	wild Prunus	Romans	Les Balmes	E585	45	7	38	3	6.25	3	09/04/2010
VA	psyllid	wild Prunus	Plaine de Valence	Beauvallon	E586	76	36	40	3	3.26	3	09/04/2010
VA	psyllid	wild Prunus	Plaine de Valence	Beauvallon	E587	25	18	7	0	0.00		09/04/2010
VA	psyllid	wild Prunus	Plaine de Valence	Beauvallon	E588	11	8	3	1	9.09	1	09/04/2010
VA	psyllid	wild Prunus	Mirmande	Mirmande	E589	40	36	4	0	0.00		09/04/2010
VA	psyllid	wild Prunus	Mirmande	Mirmande	E590	31	27	4	0	0.00		09/04/2010
VA	psyllid	wild Prunus	Mirmande	Mirmande	E591	1	1	0	1	0.00	1	09/04/2010
VA	psyllid	wild Prunus	Plaine de Valence	Gotheron	E688	18	14	4	2	8.69	2	25/03/2011
VA	psyllid	wild Prunus	Plaine de Valence	Gotheron	E689	14	8	6	0	0.00		24/03/2011
VA	psyllid	wild Prunus	Plaine de Valence	Gotheron	E690	2	2	0	0	0.00		26/03/2011
VA	psyllid	wild Prunus	Plaine de Valence	Gotheron	E691	4	3	1	0	0.00		26/03/2011
VA	psyllid	wild Prunus	Plaine de Valence	Gotheron	E692	2	2	0	0	0.00		26/03/2011
VA	psyllid	wild Prunus	Plaine de Valence	Gotheron	E693	1	1	0	0	0.00		14/04/2011
VA	psyllid	wild Prunus	Plaine de Valence	Gotheron	E694	1	1	0	0	0.00		14/04/2011
VA	psyllid	wild Prunus	Plaine de Valence	Gotheron	E695	5	3	2	1	0.00	0	14/04/2011
VA	psyllid	wild Prunus	Plaine de Valence	Gotheron	E698	10	8	2	0	0.00		15/03/2011
VA	psyllid	wild Prunus	Plaine de Valence	Gotheron	E699	23	1	22	0	0.00		16/06/2011
				STATISTICS	N	28	28	28	28	28	15	
					∑	550	305	245	24		22	
					min	1	1	0	0	0.00	0	
					max	76	36	40	3	50.00	3	
					mean	19.64	10.89	8.75	0.86	4.36	1.47	
					SE	3.53	2.35	2.28	0.20		0.24	
**Region**	**Compartment**	**Host plant**	**Sub-region**	**Locality**	**Plot codename**	**N cumulative sampling**	**N per date**		**#ESFY+**	**%ESFY+**	**#IMP**	**Sampling date**
VA	orchard	Prunus armeniaca	Romans	Romans	P40	2	2		2		2	18/02/2010
VA	orchard	Prunus armeniaca	Romans	Romans	P41	102	102		0		0	18/11/2010
VA	orchard	Prunus armeniaca	Romans	Saint-Bardoux	P42	123	97		9	9.28	30	18/11/2010
VA	orchard	Prunus armeniaca	Romans	Saint-Bardoux			26		26			15/03/2011
VA	orchard	Prunus armeniaca	Plaine de Valence	Beauvallon	P43	111	5		5		17	18/02/2010
VA	orchard	Prunus armeniaca	Plaine de Valence	Beauvallon			100		6	6.00		18/11/2010
VA	orchard	Prunus armeniaca	Plaine de Valence	Beauvallon			6		6			15/03/2011
VA	orchard	Prunus armeniaca	Plaine de Valence	Etoile-sur-Rhône	P44	6	6		6		6	18/02/2010
VA	orchard	Prunus armeniaca	Mirmande	Mirmande	P45	8	8		8		8	18/02/2010
VA	orchard	Prunus armeniaca	Mirmande	Mirmande	P46	2	2		2		1	18/02/2010
VA	orchard	Prunus armeniaca	Mirmande	Mirmande	P47	152	150		4	2.67	3	19/10/2011
VA	orchard	Prunus armeniaca	Mirmande	Mirmande			2		0			15/03/2011
VA	orchard	Prunus armeniaca	Mirmande	Villes-sur-Auzon	P48	14	14		14		0	18/02/2010
VA	orchard	Prunus armeniaca	Plaine de Valence	Gotheron	P49	279	8		8		52	26/10/2010
VA	orchard	Prunus armeniaca	Plaine de Valence	Gotheron			14		14			26/10/2010
VA	orchard	Prunus armeniaca	Plaine de Valence	Gotheron			244		47	19.26		29/03/2011
VA	orchard	Prunus armeniaca	Plaine de Valence	Gotheron			13		5			16/06/2011
VA	orchard	Prunus armeniaca	Plaine de Valence	Etoile-sur-Rhône	P50	3	3		3		3	03/03/2009
				STATISTICS	N	11			18	4	11	
					∑	802			165		122	
					min	2			0	2.67	0	
					max	279			47	19.26	52	
					mean	72.91			9.17	9.30	11.09	
					SE	27.08			2.66	3.58	4.92	

Supplementary Table S2. Contingency table of the frequency distribution of all *imp* genotypes successfully sequenced in each ecological compartment (bush, psyllid, and orchard) and each of the three growing regions.

Genotype	PO			BR			VA				
	Bush	Psyllid	Orchard	Bush	Psyllid	Orchard	Bush	Psyllid	Orchard	∑	%
I01	46	28	128	8	5	82	61	6	74	438	44.2
*I01-104**			5				1			6	0.6
*I01-248**									1	1	0.1
I01-339					1					1	0.1
I04	5	4	17	6	1	13	2	1	5	54	5.4
I04-09									1	1	0.1
*I04-175**						1				1	0.1
*I04-407**						1				1	0.1
I04-453						1				1	0.1
I03							1			1	0.1
I09	37	8	55	71	7	34	48	12	25	297	30.0
I10	6	3	8	5		15	1	3	15	56	5.7
*I10-307**			1							1	0.1
I10-310*			1							1	0.1
I11	25	12	32	6	1	3	3			82	8.3
I11-267			1							1	0.1
I13	4	3	19	3	4	14			1	48	4.8
	123	58	267	99	19	164	117	23	122	991	100

Supplementary Table S3. Contingency table displaying the frequency distribution of the six major *imp* genotypes in each ecological compartment (bush, psyllid, and orchard) and each of the three growing regions of the study.

Genotype	PO			BR			VA			
	Bush	Psyllid	Orchard	Bush	Psyllid	Orchard	Bush	Psyllid	Orchard	∑
I01	46	28	128	8	5	82	61	6	74	438
I04	5	4	17	6	1	13	2	1	5	54
I09	37	8	55	71	7	34	48	12	25	297
I10	6	3	8	5	0	15	1	3	15	56
I11	25	12	32	6	1	3	3	0	0	82
I13	4	3	19	3	4	14	0	0	1	48
∑	123	58	259	99	18	161	115	22	120	975

Supplementary Table S4. Relative proportions of the six major *imp* genotypes in the three ecological compartments and the three regions.

Genotype	PO			BR			VA		
	Bush	Psyllid	Orchard	Bush	Psyllid	Orchard	Bush	Psyllid	Orchard
I01	37.4	48.3	49.4	8.1	27.8	50.9	53.0	27.3	61.7
I04	4.1	6.9	6.6	6.1	5.6	8.1	1.7	4.5	4.2
I09	30.1	13.8	21.2	71.7	38.9	21.1	41.7	54.5	20.8
I10	4.9	5.2	3.1	5.1	0.0	9.3	0.9	13.6	12.5
I11	20.3	20.7	12.4	6.1	5.6	1.9	2.6	0.0	0.0
I13	3.3	5.2	7.3	3.0	22.2	8.7	0.0	0.0	0.8
∑	100	100	100	100	100	100	100	100	100

Supplementary Table S5. Number of major *imp* genotypes sequenced for each species of the *Cacopsylla pruni* complex, all regions combined and region-by-region.

Region	Genotype	Species A	Species B
PO+BR+VA	I01	12	27
	I04	4	2
	I09	22	5
	I10	5	1
	I11	10	3
	I13	5	2
	**#A/B**	**1611**	**961**
	**#ESFY+**	**58**	**40**
	**%A/B**	**62.6**	**37.4**
	**%ESFY+**	**59.2**	**40.8**
PO	I01	7	21
	I04	2	2
	I09	5	3
	I10	2	1
	I11	9	3
	I13	2	1
	**#A/B**	**1015**	**708**
	**#ESFY+**	**27**	**31**
	**%A/B**	**58.9**	**41.1**
	**%ESFY+**	**46.6**	**53.4**
BR	I01	3	2
	I04	1	0
	I09	7	0
	I10	0	0
	I11	1	0
	I13	3	1
	**#A/B**	**291**	**8**
	**#ESFY+**	**15**	**3**
	**%A/B**	**97.3**	**2.7**
	**%ESFY+**	**83.3**	**16.7**
VA	I01	2	4
	I04	1	0
	I09	10	2
	I10	3	0
	I11	0	0
	I13	0	0
	**#A/B**	**305**	**245**
	**#ESFY+**	**16**	**6**
	**%A/B**	**55.5**	**44.5**
	**%ESFY+**	**72.7**	**27.3**

Supplementary Table S6. List of the *imp* genotypes described to date, with their GenBank accession numbers.

imp genotype	Genbank Accession n°	Reference
I01	FN600707	[35] Danet et al., 2011
I01-104	MN116709	this study
I01-248	MN116710	this study
I01-339	MN116711	this study
I01-409	MN116712	this study
I02	FN600708	[35] Danet et al., 2011
I03	FN600709	[35] Danet et al., 2011
I04	FN600710	[35] Danet et al., 2011
I04-9	MN116713	this study
I04-175	MN116714	this study
I04-407	MN116715	this study
I04-453	MN116716	this study
I05	FN600711	[35] Danet et al., 2011
I06	FN600712	[35] Danet et al., 2011
I07	FN600713	[35] Danet et al., 2011
I08	FN600714	[35] Danet et al., 2011
I09	FN600715	[35] Danet et al., 2011
I10	FN600716	[35] Danet et al., 2011
I10-307	MN116717	this study
I10-310 (=I34)	MG972433	[36] Dermastia et al., 2018
I11	FN600717	[35] Danet et al., 2011
I11-267	MN116718	this study
I12	FN600718	[35] Danet et al., 2011
I13	FN600719	[35] Danet et al., 2011
I26	FN600720	[35] Danet et al., 2011
